# Roughness and dynamics of proliferating cell fronts as a probe of cell–cell interactions

**DOI:** 10.1038/s41598-021-86684-3

**Published:** 2021-04-23

**Authors:** Guillaume Rapin, Nirvana Caballero, Iaroslav Gaponenko, Benedikt Ziegler, Audrey Rawleigh, Ermanno Moriggi, Thierry Giamarchi, Steven A. Brown, Patrycja Paruch

**Affiliations:** 1 Department of Quantum Matter Physics, University of Geneva, 1211 Geneva, Switzerland; 2 G.W. Woodruff School of Mechanical Engineering, Georgia Institute of Technology, Atlanta, GA 30332 USA; 3 Chronobiology and Sleep Research Group, Institute of Pharmacology and Toxicology, University of Zurich, Zurich, Switzerland

**Keywords:** Biophysics, Computational biophysics, Motility, Statistical physics, Biological physics

## Abstract

Juxtacellular interactions play an essential but still not fully understood role in both normal tissue development and tumour invasion. Using proliferating cell fronts as a model system, we explore the effects of cell–cell interactions on the geometry and dynamics of these one-dimensional biological interfaces. We observe two distinct scaling regimes of the steady state roughness of in-vitro propagating Rat1 fibroblast cell fronts, suggesting different hierarchies of interactions at sub-cell lengthscales and at a lengthscale of 2–10 cells. Pharmacological modulation significantly affects the proliferation speed of the cell fronts, and those modulators that promote cell mobility or division also lead to the most rapid evolution of cell front roughness. By comparing our experimental observations to numerical simulations of elastic cell fronts with purely short-range interactions, we demonstrate that the interactions at few-cell lengthscales play a key role. Our methodology provides a simple framework to measure and characterise the biological effects of such interactions, and could be useful in tumour phenotyping.

## Introduction

**Figure 1:**
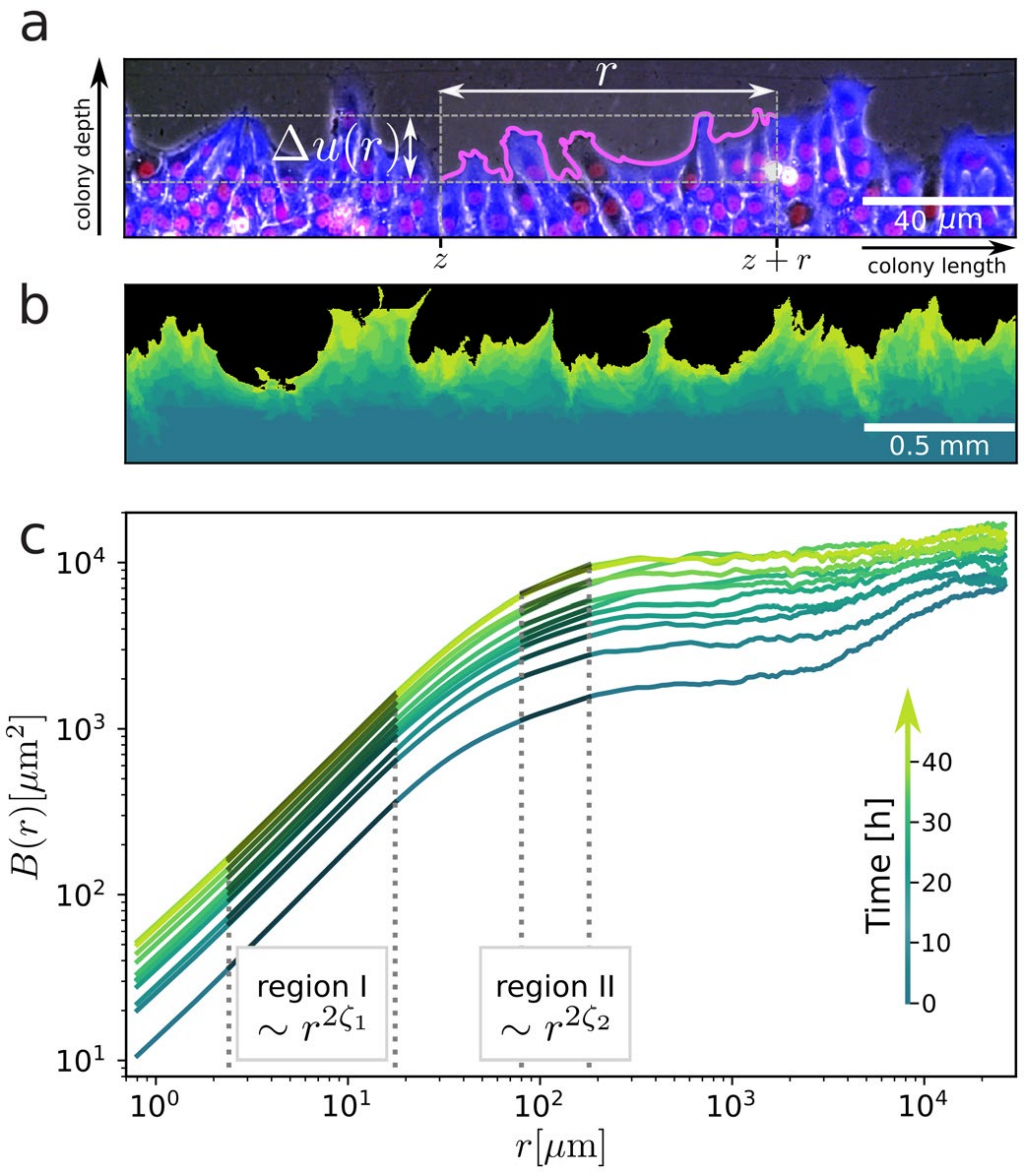
Proliferating Rat1 fibroblast cell front under control conditions. (**a**) Optical phase microscopy image of a section of the cell front, overlaid with cytoplasm (blue) and nuclei (red) fluorescence. The relative displacements $\Delta u(r{.z}) = u(z) - u(z+r)$ are measured between pairs of points separated by a distance *r*, and their correlations give a quantitative assessment of the cell front roughness. (**b**) Superposition of successive fluorescence microscopy images taken over 40 h. (**c**) Average cell front roughness $B(r) = \overline{\left<|\Delta u(r{.z})|^2\right>}\sim r^{2\zeta }$ showing two distinct regions of power law scaling. In (**b**) and (**c**) the same time scale of dark to light green is used.

The physics of elastic interfaces in disordered media provides a powerful general framework for understanding systems as diverse as eroding coastlines, propagating cracks^1^, domain walls in ferroic materials^2,3^, and proliferating cell and bacterial colonies^4,5^. In such systems, competition between the flattening effects of elasticity and the fluctuations induced by the disorder landscape leads to jerky, highly non-linear dynamics and self-affine interfacial roughening, defined by characteristic scaling exponents^6^. Formally, the geometrical fluctuations of a roughened interface are described by its monovalued transverse displacements *u*(*z*) from an elastically optimal flat configuration along the longitudinal coordinate *z*, as schematically illustrated in Fig. 1a. The scaling properties reflected in the evolution of the correlation function *B*(*r*) of relative displacements $\Delta u(r,z)=u(z)-u(z+r)$ as a function of observation lengthscale *r* are quantified by the roughness exponent $\zeta$, with

1 $$\begin{aligned} B(r) = \overline{\left<|\Delta u(r,z)|^2\right>}\sim r^{2\zeta }, \end{aligned}$$

where $\langle \cdots \rangle$ denotes an average over *z*, and ${\overline{\cdots }}$ an average over disorder realizations, when appropriate.

In physical systems, the values of the scaling exponents have been clearly related to the dimensionality of the system and the universality class of the disorder^6–8^, linked to specific types of material defects^2,9,10^. In living systems, however, where understanding cellular proliferation and migration is a crucial first step to modelling biologically important processes such as wound healing, tumour growth, and morphogenesis, the situation is far less clear, with a great richness and complexity of interactions at both sub- and super-cell lengthscales making such assignments difficult.

At both the level of single cells and colonies, the mechanical role of the cytoskeleton and cell–cell junctions has long been recognised^11–13^, forming a continuous network transferring forces between cells and through the substrates^14–16^. Growth pressure exerted by continued cell division has also been observed to drive aspects of morphogenesis^17^ and colony motion^18^, in some cases competing with the effects of ‘leader’ cells to give rise to highly heterogeneous proliferation of the cell front in scratch assay measurements^14,19^. In addition, chemical signalling between cells, for example through gap junctions or juxtacrine singalling molecules^20,21^, provides further pathways for short to medium-range interactions.

Previous studies of cell front roughening have observed fractal dimension $D_f = 1.25 \pm 0.05$, and a roughness exponent of 0.85 in propagating linear cell fronts and $0.5 \pm 0.05$ on propagating circular colonies, exhibiting a velocity of between $12\,{\text{to}}\,13.5{\mu}$m/h^22,23^. However, even with pharmacological modulation to target specific types of cellular interactions, the continued change in the shape and size distribution of the Vero cells used in these experiments, as well as their evolution from a 2D to a 3D colony, makes it very difficult to identify the effects of potentially quite different hierarchies of interactions at different lengthscales. To better explore cell front geometry at both sub-cell and super-cell lengthscales, and compare it to existing theoretical models of interfacial roughening^6^, as well as numerical simulations of growing cell colonies^24,25^, a simpler model system of a purely 2D proliferating colony of homogeneous cells would be useful.

Here, we report on the roughness and dynamics of such a 2D system: propagating Rat1 fibroblast cell fronts studied in an in-vitro scratch assay over multiple orders of lengthscales (from 1 μm to 2 cm) and several days, with and without pharmacological modulation targeting cell division rate, cell motility and mechanical intracell and intercell force transmission, as well as certain types of cell–cell communication. We find two different regimes of power law scaling in the cell front roughness, with distinct values of the exponent: below the average cell size $\zeta \approx 0.58$ for all conditions, and between 5 to 10 cells, $\zeta$ varies from 0.13 to 0.25, with a marked effect of pharmacological modulation. At the same time, these inhibitors also have significant effects on cell front dynamics, for example increasing proliferation speed when cell–cell communication via gap junctions is perturbed, and decreasing proliferation speed when cell division is repressed.

We compare our experimental observations to numerical simulations using a vertex model developed to reproduce the elastic response of epithelial sheets^24,25^, based on the energy balance between optimal cell area, cell membrane elasticity, and cell–cell adhesion. We construct a “phase diagram” of cell front proliferation and roughness under a wide set of model parameters and demonstrate that while a single region of power law scaling with relatively high $\zeta \sim 0.74$ values can be reproduced using only elasticity and nearest-neighbour interactions, the appearance of a second region with with lower $\zeta$ cannot. These results suggest that the geometry of propagating cell fronts is governed by two different hierarchies of interactions. Roughening at sub-cell lengthscales is consistent with a description based on cell membrane elasticity and short range interactions, but at few-cell lengthscales a decreased roughening points to the importance of collective interactions extending beyond nearest neighbours.

## Results

### Experimental set-up for cell front analysis

Cell fronts were prepared from Rat1 fibroblasts, modified to express green fluorescent protein (GFP) in their cytoplasm and with their nuclei fluorescently marked using Hoecht stain H33258. Individual, initially flat fronts were created by lifting off a silicone insert from confluent plates of cells, and these fronts were imaged every 4 h for more than two days using a motorised stage fluorescent microscope, with a resolution of 0.8 μm, as detailed in Methods A. After image processing of the full scratch assay panorama of 6.0 cm (see Supplementary Information (SI) 2), we extract the spatial position of the cell front $u_{front}(z)$, and approximate it by a univalued function *u*(*z*) to counter the effects of overhangs, as described in SI 3. Calculating the roughness function $B(r) = \left<|\Delta u(r{.z})|^2\right>$ for each front allows us to extract the roughness exponent $\zeta$ and its evolution with time, while the average displacement of the front $\left<u(z)\right>$ with respect to the initial position allows us to extract the front dynamics. Both the roughness and dynamics are highly reproducible for a given set of conditions, as discussed in SI 5. Where possible, *B*(*r*) and $\left<u(z)\right>$ are averaged over measurements carried out independently on different cell fronts.

### Cell fronts under control conditions

To establish the generic behaviour of the cell fronts, we first measured them under control conditions with no pharmacological modulation. As can be seen in Fig. 1b, the cell fronts evolve over 40 h from a flat initial configuration (dark green) towards an increasingly rough geometry (light green) characterized by multiple protrusions and semicircular cavities. Some cavities, like the one in the left of the image, appear to act as local pinning sites with relatively little front displacement from the initial position compared to the surrounding regions. While we do observe overhangs, most of the cell fronts present a univalued position function. We note that mitosis events, visible via the characteristic ‘bowtie’ shape of the mitotic spindle in the nuclear fluorescence channel, appear to occur at a distance of at least a few cells in from the front, but not in the cells directly at the front.

**Figure 2:**
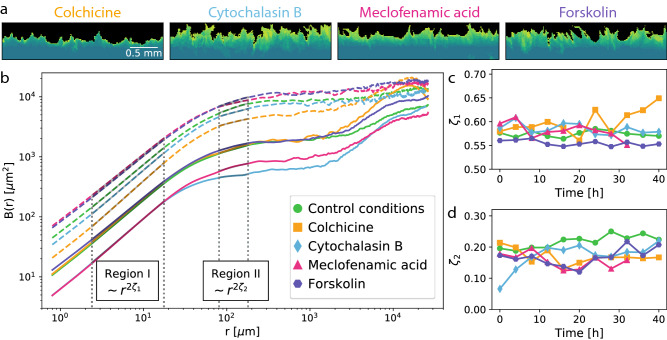
Proliferating Rat1 fibroblast cell fronts after pharmacological modulation. (**a**) Superposition of successive fluorescence microscopy images taken over 40 h, with earlier front pictured in dark green and more recent ones in lighter green. (**b**) Cell front roughness *B*(*r*) at 0 and 24 hfor the four different inhibitors, showing again two distinct power-law scaling regions. (**c**, **d**) Roughness exponents $\zeta$ as a function of time for cell fronts under control conditions (green) and after exposure to cytochalasin-B (blue), colchicine (orange), meclofenamic acid (magenta), and forskolin (purple) at sub-cell lengthcales (**c**, $\zeta _1$) and few-cell lengthscales (**d**, $\zeta _2$).

The increasing front roughness is reflected in the increasing value of *B*(*r*) at all lengthscales as a function of time, as shown in Fig. 1c, more marked in the first 12 h, then slowing to reach an apparent steady state after about 30 h (further discussed in SI 6). From a sliding analysis of an uncertainty-weighted fit of *B*(*r*) in windows of equivalent size on a logarithmic scale (as detailed in SI 7, we identify two distinct regions of power-law scaling, also observed in the power law scaling of the higher central moments of the probability distribution function of the relative displacements (as detailed in SI 8. In region *I*, for sub-cell lengthscales between 2.4 and 18 μm, we find a roughness exponent value of $\zeta _1\approx 0.58$, which remains essentially constant with time (Fig. 2c). In region *II*, for few-cell lengthscales between 80 and 180 μm, we observe significantly lower values of the roughness exponent $\zeta _2\approx$ 0.20–0.25, increasing slightly as the front proliferates (Fig. 2d). At high lengthscales above 300 μm, *B*(*r*) appears to saturate. While initially this saturation can be attributed to the globally flat configuration imposed by the experimental setup (with occasional artefacts related to the slight curvature of the patterning insert, as detailed in SI 4), once a higher value *B*(*r*) steady state is reached, saturation suggests a physical upper limit on the power law growth of cell front roughness.

**Figure 3:**
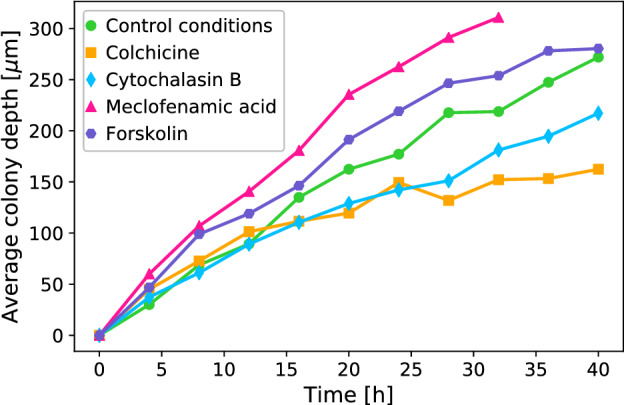
Dynamics of proliferating Rat1 epithelial cell fronts. Average colony depth as a function of time, showing the speed of proliferation is strongly affected by pharmacological modulation.

Dynamically, the front shows a linear progression of displacement from its initial position, as can be seen in Fig. 3. The average colony depth is obtained by averaging the front position over its full length and comparing this to the average initial position of the front. We measure an average front velocity of 6.5 μm/h.

### Pharmacological effects on the roughness and dynamics of cell fronts

To probe the contributions of different cellular signaling pathways to cell–cell interactions at different lengthscales, we next compared the cell fronts proliferating under control conditions to cell fronts modulated using a selection of pharmacological agents. Colchicine, which binds to tubulin and thus inhibits microtubule polymerisation (in particular crucial for spindle formation during mitosis)^26^, was chosen to target cell division rates, thus decreasing associated growth pressure in the cell colony. Cytochalasin B, which disrupts the polymerisation and network build-up of actin filaments^27^, was chosen to inhibit both cell motility and the transmission of mechanical forces between neighbouring cells. Meclofenamic acid (MFA) inhibits gap junctions communication^28^ and was chosen to disrupt this pathway of electrochemical signal transmission, previously shown to propagate logarithmically to neighboring cells up to ten neighbors distant^29^. Finally, forskolin, which activates adenyl cyclase thereby creating the signalling molecule c-AMP, an upstream activator of diverse stress and growth signalling pathways^30^, was chosen in order to globally affect cell metabolism. Confluent cell cultures were incubated with each inhibitor after lift-off patterning to establish the cell front, at concentrations reported by others and verified by toxicological titration experiments in our laboratory (see SI 1), then imaged an analysed identically to the cell fronts under control conditions.

We observe that already with a visual inspection of the cell front morphology from fluorescence microscopy images (see Fig. 2a, effects of the inhibitors can be discerned. Compared to control conditions, the colchicine-treated front shows overall much less proliferation, moving very slowly but coherently, with the cells appearing to stay in close contact to each other, and forming very limited protrusions. The MFA- and forskolin-treated fronts appear to proliferate more. We observe that forskolin also appears to strongly affect the shape of the cells, and the resulting front shows higher aspect ratio protrusions, present already in the initial configuration. As the front proliferates, these protrusions elongate in some parts forming few-cell-length fingers, separated by regions which appear to move very little from their initial position. The MFA-treated front, in contrast, is visually comparable to the control conditions front, although with slightly smaller protrusions. The cytochalasin B-treated front forms short spiky protrusions but at higher density, with an apparently more ‘localised’ pinning effect, and appears to move in a more incoherent fashion, laterally as well as forward, resulting in a higher number of overhangs.

Quantitatively, the inhibitor effects can be seen in the variations of the roughness *B*(*r*) for the different fronts, shown in Fig. 2b both at initialisation ($t=0$ datasets, shown in solid lines) and after evolution ($t= 24$ h datasets, shown as dashed lines). While the initial roughness can be to some degree affected by the execution of the lift off step (in particular through the presence of occasional rounding artefacts at high length scales, see SI 4), its values are highly reproducible between different fronts under the same conditions (as can be seen in SI 5). To evaluate the inhibitor effects is is especially important to consider also how this initial configuration evolves with time. Initially, the MFA- and forskolin-treated fronts appear to have the lowest roughness, but after 24 h show the highest roughness values. The cytochalasin-B-treated front, initially showing the lowest *B*(*r*) values, also roughens much more significantly with time. The colchicine-treated front, in contrast, initially showing the highest *B*(*r*) values, evolves more slowly than the others, and after 24 h is the least rough of the fronts.

Interestingly, while the effects on the magnitude of *B*(*r*) are quite strong, overall much less variation is seen in the values of the roughness exponents. In region *I*, in particular, $\zeta _1$ values are almost identical in the initial configuration for all the fronts, varying between 0.55 and 0.6, and little change is observed as the front evolves with time, except perhaps for the colchicine-treated front, whose $\zeta _1$ exponent appears to increase to 0.65 after 40 h of front proliferation, but also presents the highest variability. At few-cell lengthscales in region *II*, we find that fronts exposed to inhibitors generally show lower $\zeta _2$ values than fronts under control conditions. Also, while for the control condition front $\zeta _2$ evolves very slightly and approximately continuously from 0.20 to 0.25 over the 40 h experiment duration, for the cytochalasin-B-treated front, a significant increase in $\zeta _2$ from 0.07 to 0.2 occurs in the first 10 h, after which time the exponent value remains stable within the error bars. With MFA, colchicine and forskolin, the variations appear to be mostly within the error bars, although with potentially a small decrease of $\zeta _2$ values from 0.2 to 0.15 over the first 20 h.

The most significant effects of the inhibitors are observed in the dynamics of the fronts, as can be seen in Fig. 3. Both the colchicine- and cytochalasin-B-treated fronts show a marked decrease in proliferation rates, down to average velocities of 3.8 μm/h and 5 μm/h, respectively. Conversely, the front treated with MFA shows strongly increased proliferation, with an average velocity of 10 μm/h, and the forskolin front a slight increase to 7.2 μm/h.

### Numerical simulations: the importance of cell–cell interactions

To better understand the role played by cell–cell interactions in the roughness and dynamics of the observed cell fronts, we performed numerical simulations using the vertex model implemented by the *epicell* package, chosen for its capability to reproduce the mechanical response of epithelial cell sheets^24,25^.

**Figure 4:**
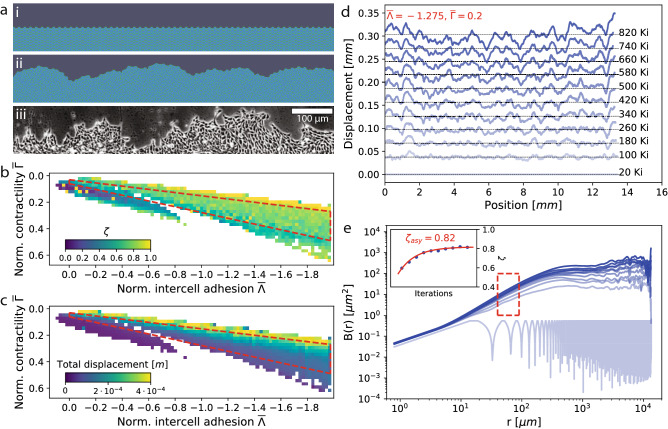
Numerical simulations of propagating cell fronts. (**a**) Initially flat fronts of regular two-dimensional hexagonal cells (i) were allowed to evolve under energy balance conditions^24^ to a steady state rough configuration (ii), visually comparable to the Rat1 fibroblast cell fronts imaged by phase contrast (iii). Phase diagram of the (**b**) roughness exponent $\zeta$ and (**c**) total displacement of the cell front simulations as a function of the normalized intercell adhesion ${\overline{\Lambda }}$ and normalized cell contractility ${\overline{\Gamma }}$. The lower branch corresponds to pathological scenarios in which little cell division occurs, accompanied by negligible front motion and roughening. The upper branch corresponds to non-pathological scenarios in which the colony grows and the front proliferates to reach an apparent steady state rough configuration and linear velocity. White space corresponds to unsuccessful simulations (see SI 10). The dashed red-coloured bounding box identifies the main region of stability and physical behaviour. (**d**) Evolution of a full $\approx 14\,\hbox {mm}$ simulated cell front for ${\overline{\Lambda }}=-1.275,{\overline{\Gamma }}=0.2$ typical of the upper non-pathological branch, progressively roughening as a function of iterations from the initial flat configuration. Selected fronts are shown at intervals of 80000 iterations (80 Ki), demonstrating a linear velocity of the interface. (**e**) Average roughness $B(r) = \overline{\left<|\Delta u(r,z)|^2\right>}\sim r^{2\zeta }$ of the fronts in (**d**), evolving from a periodic function reflecting the initial configuration to a steady state presenting a single region of power law scaling. The evolution of the roughness exponent $\zeta$, extracted by fitting the region indicated by the red box, is shown in the inset.

The Hamiltonian approach implemented in *epicell*, with

2 $$\begin{aligned} H = \sum _{cells\,\,\alpha }\frac{1}{2} K_{\alpha }(A_\alpha -A_\alpha ^0)^2 + \sum _{cells\,\,\alpha }\frac{1}{2}\Gamma _{\alpha } L_\alpha ^0 + \sum _{edges \,\,e_{i,j}}\Lambda _{i,j}L_{i,j} \end{aligned}$$

is based on energy minimisation with a balance between the cell area elasticity determined by the area elasticity coefficient $K_{\alpha }$, cell perimeter contractility determined by $\Gamma _{\alpha }$, and line tension along cell–cell junctions given by $\Lambda _{i,j}$, further detailed in SI 8. The result of such an approach is shown in Fig. 4a, with an initially regular two-dimensional hexagonal cell arrangement (i) evolving as a function of time—through cell divisions and relaxation—to a steady-state configuration (ii) with a qualitative geometry that is comparable to experimental observations of Rat1 cell fronts (iii).

We chose the simulation parameters to reflect the physics of the observed system: optimal cell size was set to the average cell size of the Rat1 fibroblasts, and the cell area elasticity was taken from previous results reproducing experimentally observed mechanical strain in cell cultures^24^. As these model parameters govern the specific physics of the system, we explored a large range of normalized cell contractility ${\overline{\Gamma }}$ and normalized inter-cell adhesion ${\overline{\Lambda }}$ parameter combinations, yielding phase diagrams such as those shown in 4b, c. White space corresponds to unsuccessful simulations, caused either by unphysical parameters or lack of numerical convergence. Two branches are apparent in all phase diagrams. The lower branch generally exhibits aberrant behaviours, such as limited cell division, cell sheet contraction instead of colony growth, and negligible front motion and roughening. The upper branch is generally well-behaved, and corresponds to normal behavior where the culture grows and the front proliferates, reaching a steady state roughness over time. We therefore defined an area of stability and physically relevant behaviour, bounded by the dashed red box indicated on the phase diagrams, within which we further analysed the evolution of the cell front roughness.

Each successful simulation was run for 12 h or 20,000 divisions, and then analysed individually, as illustrated in Fig. 4d for the full $\approx$ 14 mm simulated cell front with ${\overline{\Lambda }}=-1.275,{\overline{\Gamma }}=0.2$, corresponding to a typical non-pathological behaviour. The front position was extracted at regular time intervals, here 80,000 iterations (80 Ki), and shows a constant proliferation velocity as well as visibly steady-state roughness configuration at the final time steps, usually after 500 Ki. The average roughness *B*(*r*) was computed at each time step, starting with a highly correlated behaviour due to the initial front periodicity. Since the cells in the simulation are flat-sided polygons, roughness at sub-cell lengthscales has no biophysical relevance. As a function of time, *B*(*r*) generally tends towards a steady state behaviour with power law scaling at low lengthscales, and a transition towards a roughness saturation at around 0.5 mm.

The roughness exponent $\zeta$ was extracted as in the in-vitro experiment from the region highlighted by the red box shown in Fig. 4e. Last, the evolution of $\zeta$ as a function of time was fitted by an exponential function to extract the asymptotic value of the roughness exponent $\zeta _{asy}$.

The phase diagram of asymptotic roughness exponents for each set of conditions can be seen in Fig. 4b, and shows a large region of uniform value in the stable branch bounded by the red dashed box. As discussed above, this region corresponds to non-pathological cases, with the corresponding total displacement in Fig. 4c increasing both with lower contractility as well as lower inter-cell adhesion. The mean exponent within this region ${\overline{\zeta }} = 0.74$, with standard deviation $\sigma = 0.09$.

The overall behaviour of the cell front simulations seems to suggest that power law scaling with relatively high $\zeta$ values can be reproduced using a simple Hamiltonian based on mechanical properties and nearest-neighbor inter-cell coupling. Although the model includes no external disorder, the simulations produce some internal disorder, and the values of $\zeta$ we obtain are in the same range as for example those of the so called random bond disorder universality class for which $\zeta = 2/3$. However, the simulations fail to capture the experimentally observed lower roughness exponent values, and of course the presence of two distinct scaling regimes. These results imply that the geometry of in-vitro propagating cell fronts is governed by two different hierarchies of interactions. The experimentally observed roughening at sub-cell lengthscales can plausibly be described as a balance of cell membrane elasticity and short range interactions. However, at few-cell lengthscales, collective interactions appear to be necessary to decrease roughening and establish a second region of power law scaling with lower values of the roughness exponent.

## Discussion

Our measurements of pharmacologically modulated cell fronts point to a complex interplay of interactions at multiple scales, and between static and dynamic parameters. Unsurprisingly, cell division appears to be a strong driver of front movement, in agreement with past observations in other systems^29^. At intermediate length scales of 5–10 cells, those modulators that promote cell mobility or division also lead to the most rapid evolution of cell front roughness. For example, inhibiting cell division with colchicine results in smaller changes of *B*(*r*) at intermediate lengthscales (5–10 cells) over time. In reverse, inhibiting cell–cell communication via gap junctions appears to both significantly promote cell front mobility and leads to the most rapid evolution of cell front roughness *B*(*r*) with time. Thus, our results also suggest that communication across these intermediate lengthscales may play an important role in determining cell front movement, and by inference biological processes such as tumor invasion, wound healing, and tissue development. We note that dye diffusion assay studies of gap junctions networks in rat kidney cells^29^ have shown characteristic effective concentration lengthscales of the order of 200 μ, in the range where we observed the second power law growth region of *B*(*r*). Forskolin, activating the same cellular pathways as the most common class of G protein-coupled receptors responding to diffusible extracellular ligands, affects *B*(*r*) in the same range.

At longer lengthscales ($1000\,{\mu}m$) where we observe the cell front flattening over time, we propose that these changes might arise from specific growth behaviors and not as simply an artefact of the initially defined flat front configuration. Indeed, while *B*(*r*) continues to increase with time, as can be seen in Fig. 1, this flattening becomes more, rather than less apparent. Overall, when cavities are present in the front, instead of proliferation globally in the direction perpendicular to the line of the front, the cells appear to actively redirect and flow towards the cavity, filling it and effectively ‘smoothing’ the front geometry at high length scales, as can be seen in SI 9. The specific physical or biological determinants of proliferation region and direction remains a fascinating question meriting further study.

Finally, at short lengthscales of less than two cells, from the analysis of the numerical simulations we conclude that purely elastic and short range interactions lead to a universal power law scaling behaviour of the cell front roughness. The observed high values of the roughness exponent are in line with those seen for known universality classes of disordered elastic systems with short range interactions, such as one-dimensional interfaces subjected to thermal fluctuations ($\zeta _{th} = 0.5$) or random bond ($\zeta _{RB}=2/3$) disorder^6,31^. In-vitro, the sub-cell roughness scaling regime, with $\zeta _1 \sim 0.58$ is also coherent with such a description. However, the simulations fail to reproduce the lower roughness exponent value observed experimentally at few-cell lengthscales. This failure reinforces our hypothesis that a hierarchy of interactions is necessary to fully capture the behaviour of proliferating cell fronts, and that mid-lengthscale interactions are particularly important.

More generally, our approach extends literature applying models initially derived for physical systems to biological ones, demonstrating that many aspects can be described in purely physical terms. Defining different scaling exponents for interfacial roughening at different lengthscales during normal cell growth, and linking the evolution of these exponents to specific cellular pathways, represents a novel approach to understand the origins of pathological growth in deleterious situations, including cancer and traumatic injury.

### Supplementary Information

Supplementary Information.

## Data Availability

The experimental and numerical data that support the findings of this study are openly available in Yareta at http://doi.org/10.26037/yareta:eyj33z7alje35cypydi5tecb2q in compliance with SNSF Data Management Plan guidelines.

## Appendix 1

### Experimental methods

#### Cell preparation

Rat-1 fibroblasts were maintained in Dulbecco’s Modified Eagle Medium (DMEM) with high glucose, L-glutamine, and phenol red (*Sigma-Aldrich*, Darmstadt, Germany #D5796), supplemented with 1% penicillin/streptomycin (*Sigma-Aldrich*, Darmstadt, Germany, #P4333) and 10% fetal bovine serum (FBS) (*Gibco Thermo Fisher*, Reinach, Switzerland, #10270106). Rat-1 fibroblasts were cultured at 37 °C in a humidified incubator with 5% $CO_2$ and were passaged 1:6 when they reached  80% confluence after washing with PBS and detaching with trypsin 10x (*Sigma-Aldrich*, Darmstadt, Germany #15400054). At the final passage, the cells were seeded around a prepositioned 6 × 2 cm^2^ insert (*Ibidi*, Gräfelfing, Germany) and incubated to confluence. The insert was removed and the resulting front allowed to settle and set for 2 h prior to the first imaging steps.

GFP fluorescence marking of the cytoplasm was realised through retroviral modification using V21, premade LV vector, (vesicular stomatitis virus (VSV) glycoprotein (G) pseudotyped) obtained from the Viral Vector Facility of the Neuroscience Center Zurich. Nuclei were fluorescently marked using 32.0 micromolar Hoecht stain (*Sigma Chemicals*, USA, H 33258) for 3 h.

For pharmacological modulation, we used (i) colchicine (*Sigma-Aldrich*, Darmstadt, Germany, #C9754), 0.32 μM concentration (ii) cytochalasine-B (Lucerna Chem AG, Luzern, Switzerland, #AGS-C-1027-5MG), 0.50 μM concentration, (iii) meclofenamic acid (same as i and ii), and (iv) forskolin (same as i and ii). Employed concentrations were established in separate titrations with each inhibitor to determine effects on cell mortality, growth and morphology, as detailed in SI 1.

#### Optical imaging

Brightfield phase and fluorescence microscopy imaging was carried out using a *Leica* DM6000 microscope coupled with a 1 Mpxs *Hamamatsu* CCD, at 1 px = 0.8 μm resolution. To obtain the full panorama of the proliferating front, a frame matrix of $3\times 80$ images, each $800\times 800$ μm^2^ was captured every 4 h, using software-controlled motorised stage steps. While in most cases the fluorescence signal remained relatively stable, for some fronts significant contrast variation artifacts made extraction of the imaged panorama extremely difficult. Such fronts, for instance the MFA-treated 36 and 40 h timepoint, were not included in the dataset for analysis.

## Appendix 2

### Numerical methods

Numerical simulations of proliferating cell fronts were carried out using the *epicell* package, developed at the University of Geneva^32^, adapted to the modeling of mechanical properties of two-dimensional cell sheets^24,25^.

Simulations were performed with the *freeBoundary* application, on a $20\times 1000$ grid of two-dimensional hexagonal cells. The model was modified to favour divisions occurring just behind the cell front, in line with our empirical observations.

The cell area elasticity parameter *K* was set to $2.256\times 10^9\,\hbox{N/m}^3$ following previous studies on mechanical properties of cell sheets, and the preferred cell area $A^0$ was set to $3.14\times 10^{-10}\,\hbox{m}^2$, following the experimental observations on the average size of the Rat1 cell line. The normalized inter-cell adhesion, ${\overline{\Lambda }}$, was explored in phase space from $-1.975$ to 0.5 in steps of 0.025. Likewise, the normalized cell perimeter contractility, ${\overline{\Gamma }}$, was explored in phase space from $-0.3$ to 0.675 in steps of 0.025.

4000 simulations were performed with a running time constraint of 12 h and a maximum division number of $10^5$. Of these, 881 simulations were successful and were used as the basis for the discussion in the main text. Unsuccessful simulations are discussed in SI 10.
