# Supplementary material for: Roughness and dynamics of proliferating cell fronts as a probe of cell–cell interactions
Source: Sci Rep. 2021 Apr 23;11:8869. doi: 10.1038/s41598-021-86684-3 (PMC8065107; doi:10.1038/s41598-021-86684-3)
Supplement: Supplementary file 1 — Supplementary Information. [file 41598_2021_86684_MOESM1_ESM.pdf]

Supplementary Information: Roughness and dynamics of proliferating cell fronts as a probe of cell-cell interactions

Guillaume Rapin[1,*,§], Nirvana Caballero[1,*], Iaroslav Gaponenko[1,2], Benedikt Ziegler[1], Audrey Rawleigh[3], Ermanno Moriggi[3], Thierry Giamarchi[1], Steven A. Brown[3], and Patrycja Paruch[1,†]

[1]Department of Quantum Matter Physics, University of Geneva, 1211, Geneva, Switzerland
[2]G.W. Woodruff School of Mechanical Engineering, Georgia Institute of Technology, Atlanta, GA, 30332, USA
[3]Chronobiology and Sleep Research Group, Institute of Pharmacology and Toxicology, University of Zurich, Zurich, Switzerland
[*]These authors contributed equally to this work
[§]guillaume.rapin@unige.ch
[†]patrycja.paruch@unige.ch

1 Pharmacological modulator titration

To determine which concentration of the inhibitors would be most effective without significantly affecting cell mortality, separate titrations were conducted for each of the compounds on fully confluent healthy Rat1 cell cultures. Starting with values commonly used in our previous experiments, the applied concentration was increased in pseudo-logarithmic steps (1x, 2x, 5x, 10x, 20x, etc), up to a killing concentration determined by visual inspection of the cells after 24 hours of exposure, in comparison with cells prepared at the same time under identical conditions without inhibitor exposure.

Inhibitor effects on cell growth, morphology, and mortality were evaluated based on the presence and extension of lamelipodia, the distance between cell nuclei, loss of confluence, and of course the amount of dead cells. For colchicine, the initial concentration of 0.32 μM did not affect cell mortality, but morphology effects of a more rounded and globular cell shape were beginning to be apparent. At higher concentrations, these morphology changes were more significant, but we also observed significant mortality increase above 0.625 μM, leading us to conclude that the original concentration of 0.316 μM was already at the optimal value. For the cytochalasin B, starting from a concentration of 0.1 μM, cell mortality increased sharply from less than 5% to more than 30% for 0.5 to 1 μM, and the colony was seriously depopulated (50% mortality at 2 μM). We therefore chose an effective concentration of 0.5 μM. The toxicity of meclofenamic acid was likewise easily decided, the mortality again increasing rapidly from less than 5% to more than 80% between 50 and 100 μM, leading us to chose an effective concentration of 50 μM. Finally, with forskolin, we did not observe significant effects on cell mortality. At 5 μM we began to see apparent elongation of the cells. These effects appeared to saturate at 10 μM, which was therefore the concentration chosen for the experiments.We generally observed that at concentrations approaching the killing concentration, cells appear to shrink and we begin to observe loss of confluence with increasing amounts of empty space between the cells. The cells also detached readily from the substrate surface, dying and floating above the rest of the colony.

2 Image processing

Each of the 1000 x 1000 px images acquired using the Leica DM6000 were indexed by the x, y position of the motorised sample stage (the vertical height z was kept constant once the desired focal plane was established), allowing us to stitch the individual images in the frame matrix into a full panorama of the proliferating front, as shown in Fig. 1(a), using an in-house developed Python algorithm. To fully discriminate the cell colony from the background the original greyscale fluorescence images are binarised using the Otsu algorithm and the brightness histogram for the panorama, as seen in Fig. 1(b), with the colony in white. As a result of varying GFP expression between cells, the binarisation still leaves some dark areas behind the cell front, necessitating a "hole filling" step carried out with the Scipy function, "binary_fill_holes" , which leads to the image shown in Fig. 1(c). In this image, we identify the cell culture as the largest group of connected white pixels, obtained using the Scikit

Forskolin	Cell mortality
2 µM	Negligible (<5%)
5 µM	Negligible (<5%)
**10 µM**	Negligible (<5%)
20 µM	Significant (30%)

Colchicine	Cell mortality
**0.32 µM**	Negligible (<5%)
0.63 µM	Significant (10%)
1.25 µM	Significant (50%)
3.20 µM	Significant (90%)

Cytochalasin B	Cell mortality
0.1 µM	Negligible (<5%)
0.25 µM	Negligible (<5%)
**0.50 µM**	Negligible (<5%)
1.00 µM	Significant (30%)
2.00 µM	Significant (50%)

MFA	Cell mortality
10 µM	Negligible (<5%)
25 µM	Negligible (<5%)
**50 µM**	Negligible (<5%)
100 µM	Significant (80%)
250 µM	Significant (80+%)

Table 1. Results of individual toxicity titrations to establish the final concentration (indicated in bold) of the inhibitors used in the study.

"label" function. The set of x, y coordinates of the front position is finally obtained using the Scikit function "find_contours", and can be seen in Fig. 1(d), overlaid on the original fluorescence microscopy image the cell colony.

3 Monovalued approximation

The extracted front position follows the contours of the individual cells, and shows small overhang features where a single cell or small clusters of a few cells at the edge of the colony orient somewhat diagonally. However, the framework of disordered elastic systems, with an analysis of the correlation function $B(r)$ of relative displacements $\Delta u(r)$, is only applicable in the case of a univalued interface, meaning that elements such as overhangs, holes, and islands cannot be treated within this approach. We therefore approximate the extracted front position by a univalued function.

This correction is carried out using 3 different approaches, as shown in Fig. 2: 'outer' contour (yellow), choosing the highest value of $u(z)$ if multiple points of the front are present at a given lateral coordinate z; 'inner' contour (green), choosing the lowest value of $u(z)$ if multiple points of the front are present at a given lateral coordinate z; and 'sum' contour (blue), adding all the pixels of the colony at a given lateral coordinate z to obtain an effective univalued $u(r)$.

As can be seen in Fig. 2(a), the roughness $B(r)$ calculated for each of these corrected fronts shows small differences at sub-cell lengthscales, but these become insignificant by the time the behaviour at few-cell lengthscales is examined. Since the sum approach effectively smooths the interface, to preserve at least partially the effect of the overhangs, which are clearly a feature of the biological system, we chose to use the outer contour for all the analysis presented in this study.

This results in abrupt but still univalued 'jumps' in the front position. In order to estimate the impact of these jumps on the front geometry, we subtracted the outer and inner contours of the front, and extracted the statistics of the resulting deviations from a straight line. As can be seen in Fig. 3(a,b), the jumps, reflecting the original overhangs, are generally limited to at most a width of 8 µm (i.e. less than the characteristic cell size) and an average height of 200 µm (i.e. approximately 10-12 cells).

4 Rounding of the initial configuration

During the lift-off patterning, the flexibility of the silicone insert used to demarcate the initial position of the cell front in a confluent cell culture can lead to a rounded arc geometry rather than a straight line configuration. This arc corresponds to a segment of a circle with a radius of approximately 1 m, which can be quite precisely overlaid over the initial front position, as shown in Fig. 4(a), and contributes an identifiable artefact to the calculation of the front roughness $B(r)$. As can be seen in Fig. 4, $B(r)$ for such a 1 m radius circle arc shows a power law growth, with a roughness exponent $\zeta = 1$. However, the initial roughness levels are over 4 orders of magnitude lower than that of the cell front itself, starting at approximately 2×10^{-4} µm^2 at $r = 1$ µm. The roughness increases to 3×10^4 at $r =$ 30mm, the highest available lengthscale of the analysis. While this artefact, when present, becomes the dominant contribution to the experimental observations above $r = 2$ mm, and affects to a lesser degree the roughness of the cell front above 500 µm, it does not affect our analysis of the double power law growth behaviour with very different roughness exponents at subcell (4–18 µm) and few-cell lengthscales (80–180 µm).

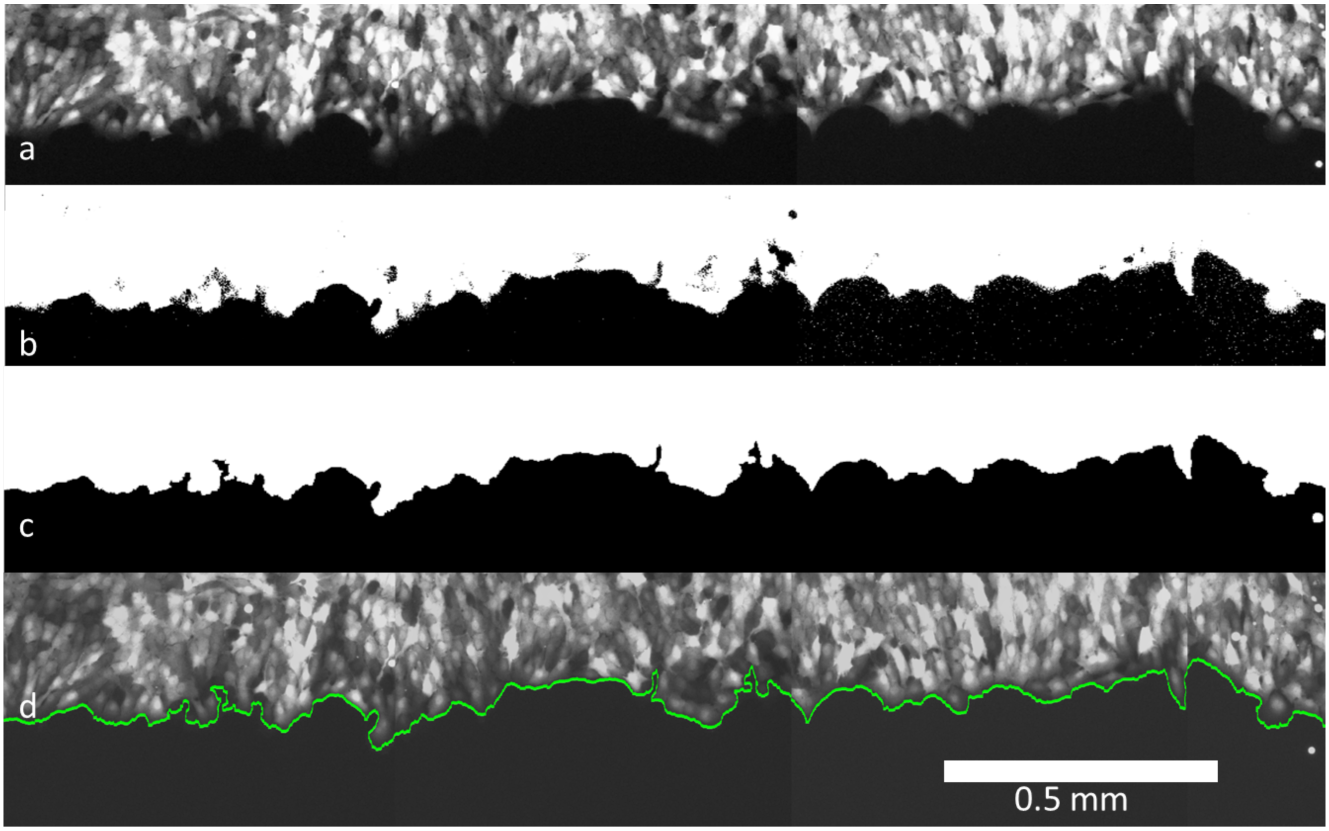

Figure 1. Image processing to obtain front position. The fluorescence microscopy image (a) is binarised using the Otsu algorithm (b), and fluorescence variations corrected with a “hole filling” step (c). The colony is identified as the largest connected island on the image and the front position (green) is extracted as its lower contour, shown overlaid on the original fluorescence image (d).

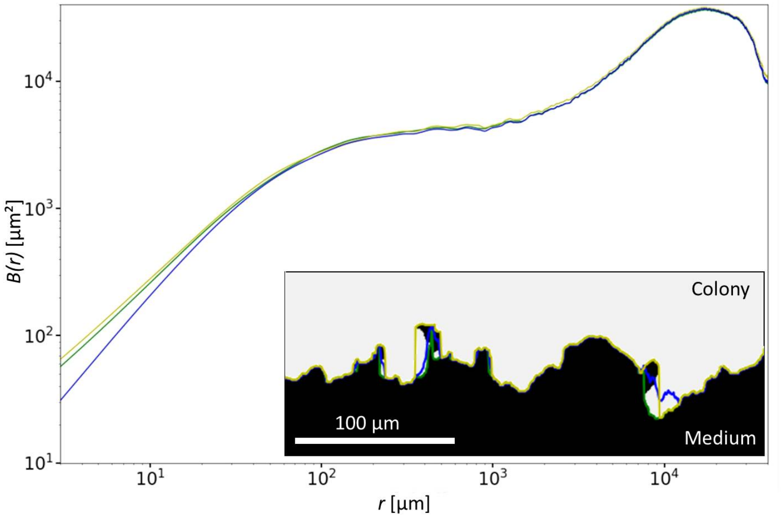

Figure 2. Univalued correction of front position. (a) Cell front roughness $B(r)$ extracted after correction of the intermittent overhangs using the ‘outer’ (yellow), ‘inner’ (green), and ‘sum’ (blue) contours shows only very minor differences at subcell lengthscales.

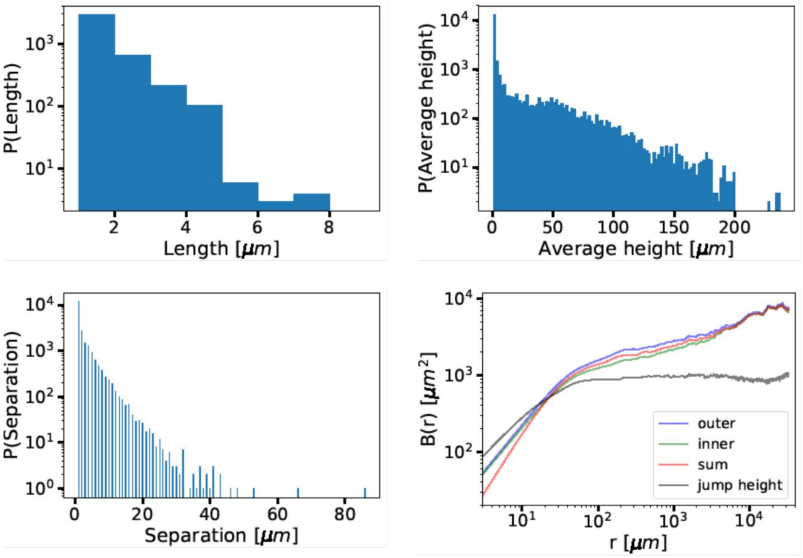

Figure 3. Statistics of jump features resulting from the correction of overhangs in the cell front position. (a) Distribution of jump widths along the cell front. No overhangs wider than approximately a half cell size are observed. (b) Distribution of average jump heights perpendicular to the cell front. (c) Distribution of the interjump separations along the cell front.

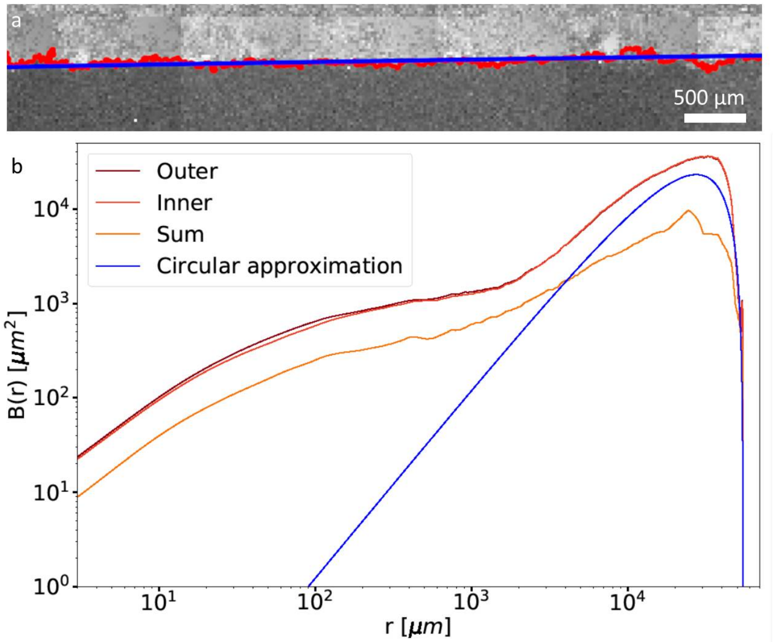

Figure 4. (a) Fluorescence images of the cell front (GFP-stained cytoplasm), with the extracted front position (red), overlaid with the circle segment approximation (blue). (b) Roughness $B(r)$ calculated for the three different overhang correction approaches, and of the circle segment used to approximate the initial position resulting from a slight bending of the flexible silicone insert.

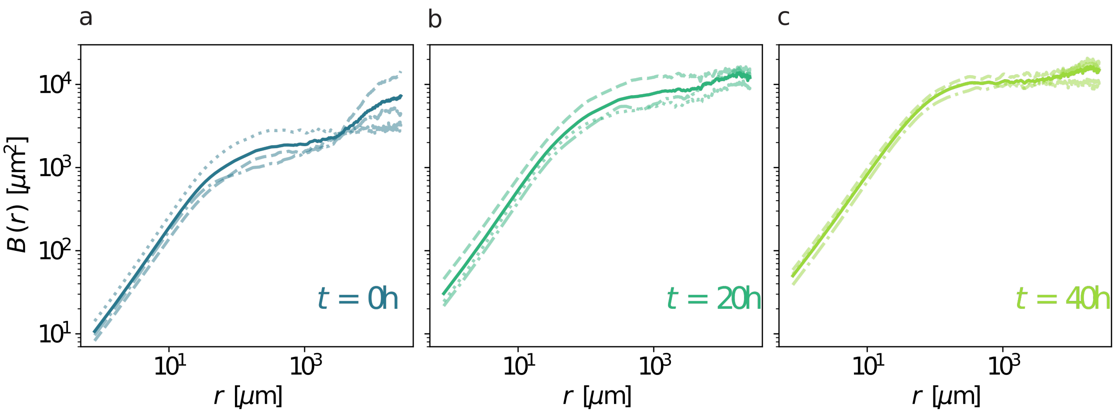

Figure 5. Reproducibility of roughness analysis. Roughness function $B(r)$ computed for three experimental realisations of the cell front under control conditions (discontinuous lines), and the average roughness (solid line) at different evolution times (a) $t = 0$, (b) 20, and (c) 40 hours.

5 Reproducibility of roughness analysis

Our previous studies of physical interfaces have shown that sufficiently long individual fronts, or sufficient different iterations of shorter fronts are necessary to accurately extract the value of the roughness exponent ζ[1,2]. The relatively large cell front panoramas obtained in our experiments (length $L \sim 2^{16}$ px or $\sim$ 5cm) provide ample statistics for the analysis of the roughness $B(r)$, restricted to $r \leq \frac{L}{2}$, and thus a high level of confidence in its accuracy.

As a further test, and to check the reproducibility of the experimental protocol, we repeated each experiment three times under control conditions to obtain the time evolution of three separate cell front panoramas over 40 hours. For each of these realizations i ($i = 1,2,3$), we obtain the functions $u_{i,t}(r)$ describing the position of the cell-front after an evolution time $t = 0,4,8,\cdots 40$ hours. We compute the roughness $B_{i,t}(r)$ for each of these fronts, and the average $B_t(r) = \frac{1}{3}\sum_{i=1}^{3} B_{i,t}(r)$. over the three realizations i at the same time t. As shown in Fig. 4, the roughness does not appear to change significantly from one realisation to the other (apart from the rounding artefact present in one of the realisations, as discussed in Sect. 4), and after a long evolution time, the roughness obtained for the three realisations appears to converge to the average value.

6 Evolution to steady state roughness under control conditions

During our observations of their proliferation the cell fronts are maintained *in-situ* under the microscope, without changing the medium or adding nutrients. We therefore limited our discussions of the time evolution of the fronts in the main paper to 40 hours, to ensure that chemical depletion did not play a role in the observed cell behaviour. However, to establish when significant cell death and deterioration of the colony does occur, we also followed a smaller set of fronts for longer periods. In pharmacologically modulated cell fronts, this occurs already at 60–70 hours, while under control conditions cell fronts appear healthy and proliferate beyond 100 hours.

The results of our longest experiments can be seen in Fig. 6(a,b), following the average position and roughness, respectively, of a single front for 112 hours under control conditions. The front proliferates with decreasing speed over the full period of observation, approximately following a logarithmic time dependence, although increased acceleration is observed at 12–18 hours, and very little proliferation appears to occur beyond 100 hours. The roughness $B(r)$ calculated at subcell lengthscales ($r = 10$ µm, blue) remains essentially constant throughout the measurement, suggesting a steady state which is already reached by the time the initial configuration of the experiment is established, and which could be expected for a roughness determined largely by intracellular interactions. At few-cell lengthscales ($r = 100$ µm, red), on the other hand, the roughness increases over the first 24 hours from an initial lower value reflecting the artificially flat configuration induced by the removal of the insert. After approximately 24–30 hours, the value of $B(r)$ appears to also reach a steady state, with no significant increase, despite the fact that the front continues to proliferate with no noticeable deterioration or cell death.

7 Extracting the roughness exponent

One of the most important features of the roughness function $B(r)$ is that it provides information about the interactions present in a system. To capture this information, two correlated elements are necessary. First, the spatial regions where the function

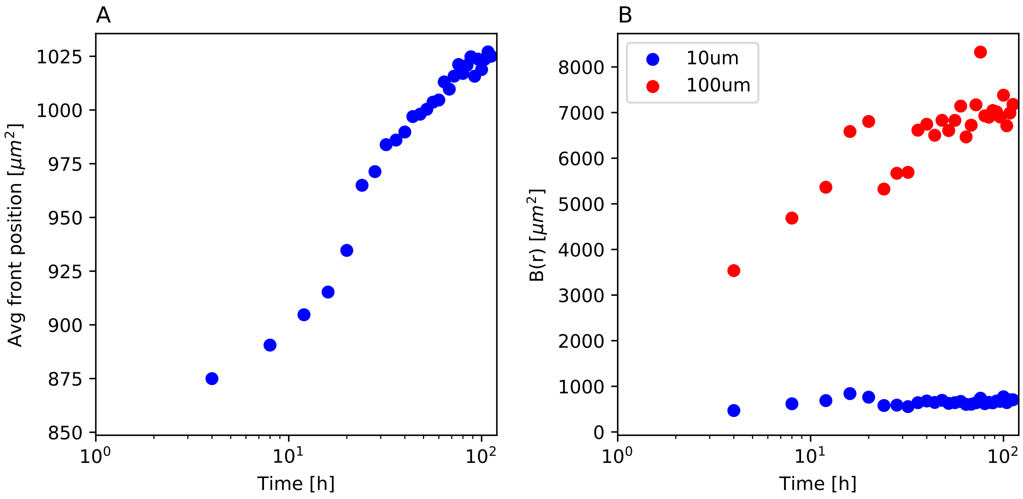

Figure 6. Evolution of a single proliferating Rat1 epithelial cell front followed over 112 hours (a) The average position of the cell front, used to track its displacement during proliferation, shows a close to logarithmic dependence as a function of time (b) The roughness $B(r)$ calculated at $r = 10$ μm (subcell) and $r = 100$ μm (few-cell) lengthscales, in blue and red, respectively. A steady state with little increase in the roughness is observed already from the initial configuration at subcell lengthscales, and beyond approximately 30 hours at few-cell lengthscales.

follows a power law need to be identified, and second, the fit of the function with a power law should be done over this region to extract the roughness exponent describing the interactions at this lengthscale.

In the theory of disordered elastic systems, where, for example, a system evolves as result of the competition between the elasticity of the interface c, the disorder or pinning, and thermal fluctuations proportional to the temperature T, it is well known that the roughness function at short lengthscales is dominated by thermal fluctuations, and follows a power law behavior $\frac{T}{c} r^{2\zeta_{th}}$, where the roughness exponent is given by the thermal exponent $\zeta_{th} = 0.5$. However, at large lengthscales $B(r)$ is dominated by disorder, if present, and depending on the disorder type follows a different power law proportional to $\sim r^{2\zeta_{dis}}$. For example, for random bond disorder the scaling is governed by $\zeta_{dis} = \frac{2}{3}$.

In the case of biological systems, the nature of the interactions between cells and how they affect the dynamics and statics of a cell front is not yet well established, and is what motivates the current study. In order to apply the theory of disordered elastic systems to effectively capture the information about the interactions in a growing colony of cells, we identify the regions where $B(r)$ follows a power law behavior as follows: we perform a weighted fit of $\log B(r)$ with a linear function of r over the region $[\log r_i, \log(r_i + w_i)]$, where r_i is the starting point and w_i is the length of the fitting region chosen according to the initial value r_i, in order to always obtain a window of equivalent size on a logarithmic scale for each starting point (see Fig. 7). The weights used for the fit are computed as the uncertainties Δy associated to each point of $y = \log B(r)$. These uncertainties can be estimated as $\Delta y = \frac{1}{B(r)} \Delta B(r)$, where $\Delta B(r) = \frac{\text{std(all observed values)}}{\sqrt{\text{number of observations-1}}}$, and the number of observations decreases when r is increased (observations=total interface length - r). For each fit we compute the goodness of the fit through R^2.

As can be seen in Fig. 8, which shows R^2 vales for fits of the roughness $B(r)$ at $t = 0$ hours averaged for the three fronts under control conditions, two regions emerge where the power law fits with $B(r) \sim r^{\zeta}$ are highly pertinent. At short lengthscales (for small r_i) we obtain very high R^2 values above 0.9998, even for relatively large window sizes w_i. R^2 then abruptly decreases, and a second local maximum with values around 0.9992 is reached at ~ 80μm. This behaviour suggests two separate power law scaling regimes are present in $B(r)$.

To choose the best window size for each region we complemented the mathematical consideration of R^2 with additional biological arguments. For $r_i = 4$μm, even if the goodness of the fit is high up to 50μm, we restricted the fit to not exceed the average length of a cell ~ 20 μm, in order to capture the behavior of $B(r)$ at subcell lengthscales, thus defining a first region $I = [4, 16]$μm. For the second region, we considered that important mechanisms of intercell communication, in a wide range of mammalian epithelial cells, have a range of at most a few cell lengths - for example the cell networks reported for gap junction communication in rat kidney cells[3] as well as mechanical interactions via the cytoskeleton and force transfer via the substrate, which were found to decrease linearly with distance[4]. We note here that the inhomogeneous distribution of cell division we observed, which seemed to be more prevalent in cells close, but not directly at the colony edge, can also be considered as an

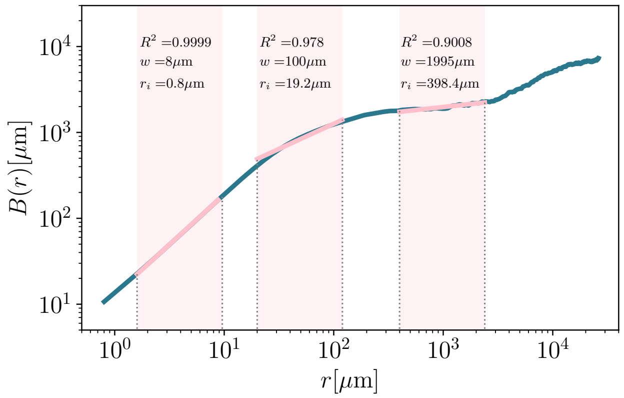

Figure 7. To determine the regions where the roughness function follows a power law behavior, we explored different ranges $[r_i, r_i + w_i]$ to fit $B(r)$. Here, 3 different power law fits of the roughness of the initial front configuration at $t = 0$ under control conditions are shown, for $r_i = 0.8, 19.2$, and $398.4\mu m$, and equivalent window-sizes on a logarithmic scale $w_i = 8, 100$, and $1995\mu m$. We measured the goodness of the fit through R^2.

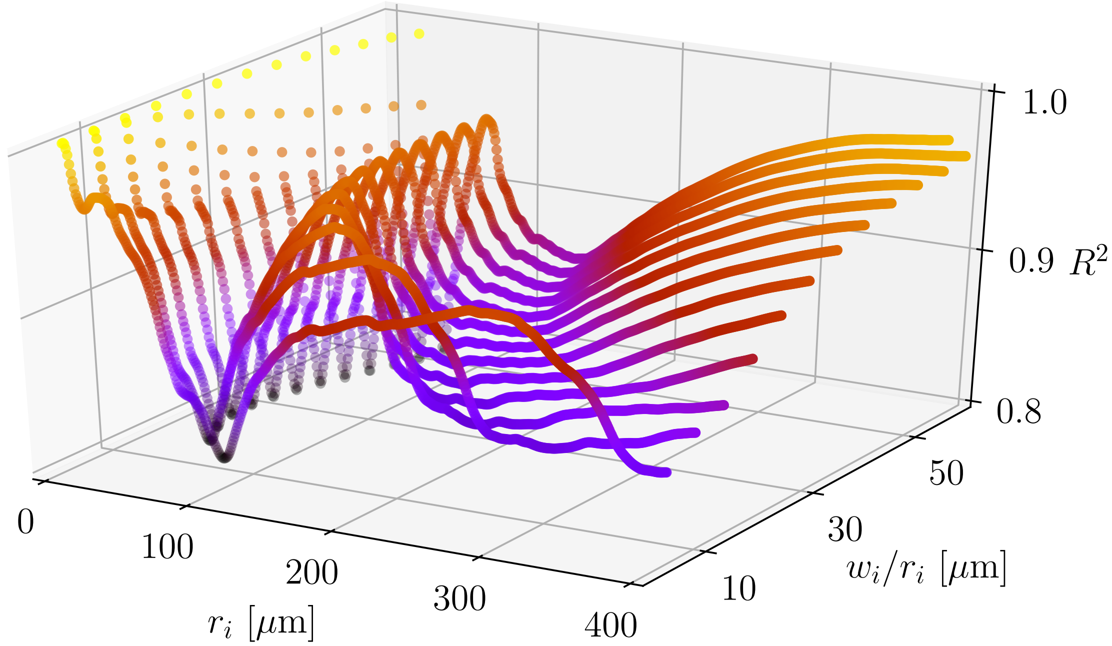

Figure 8. Identifying power law scaling regimes. R^2 obtained for the weighted fit of $\log B(r)$ averaged for the three fronts under control conditions at $t = 0$. The fit was performed over the regions $[r_i, r_i + w_i]$, where w_i was chosen for each r_i to obtain an equivalent window size on a logarithmic scale, independently of r_i. Two regions with high R^2 are observed, one at short lengthscales, and the other with a local maximum at $r_i \sim 80\mu$m, separated by a region of low r^2, suggesting two separate power law scaling regimes.

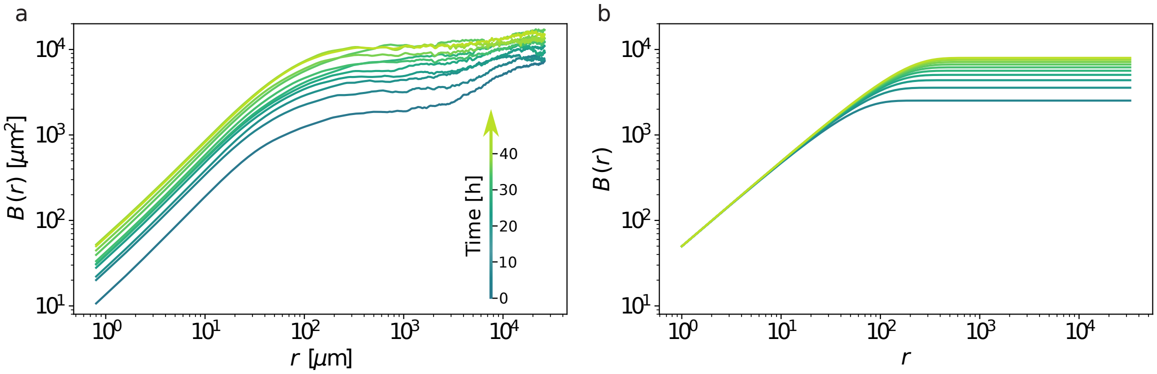

Figure 9. (a) Roughness of a cell colony under control conditions (same data as shown in Fig. 1(c) of the main paper) and (b) roughness of an elastic line evolving according to the Edwards–Wilkinson equation at time intervals of $t = 10^3$.

interaction at this range. We therefore restricted the second region to $II = [80, 180]$ µm. For all the experimental cell fronts, fits of $B(r)$ were performed over these two regions I and II to obtain ζ_I and ζ_{II}.

As a further check to ensure that the second region of power law scaling was not a spurious artefact of crossover between a single power law scaling regime and a flat function, we also compared our experimental results to simulations of specifically such a situation, an elastic line evolving in a thermal bath from an ideal flat configuration to an equilibrium rough configuration in the Edwards-Wilkinson model (E–W).

The E–W elastic line model describes the evolution of an interface $u(y,t)$ with elasticity c subject to a thermal bath of temperature T with friction η:

$$\eta \partial_t u(y,t) = c\partial_y^2 u(y,t) + \xi(y,t). \tag{1}$$

In this equation, ξ is a Gaussian white noise of zero average and correlations $\langle \xi(y_2,t_2)\xi(y_1,t_1)\rangle = 2\eta T \delta(y_2 - y_1)\delta(t_2 - t_1)$.

For an initially flat elastic line the correlations in its geometry will evolve in time as a result of the competition between the interface elasticity and the thermal fluctuations[5]. For finite times, a memory of the initial conditions remains in the roughness $B(r,t)$. As t grows correlations spread along the interface[6].

$$B(r,t) = \frac{Tr}{c}\left[1 - \frac{1}{\sqrt{\pi}zr}\left(e^{-z^2r^2} - 1\right) - \frac{2}{\sqrt{\pi}}\int_0^{zr} e^{-t^2}dt\right]. \tag{2}$$

The roughness of the elastic line is then characterized by two well-defined regimes: a power law $\frac{T}{c}r^{2\zeta_{th}}$ at short lengthscales, and a flat function at large scales. In order to check if the crossover between these two regimes could lead to a spurious second power law regime, we analyze the roughness of an elastic line described by the arbitrary chosen values $c = 1$, $T = 50$, $\eta = 1$. In Fig. Fig. 9 we show the time evolution of the roughness at time intervals of 1000 (in arbitrary units). For comparison we also show the same data as presented in Fig. 1(c) of the main paper for the roughness of a cell colony under control conditions.

We analyze the roughness of the elastic E–W line using the same approach as described above for the cell fronts. The goodness of fit R^2 for the elastic line at $t = 1000$ is shown in Fig.10, with a clear maximum observed at short lengthscales, corresponding to the expected region of power law scaling. The goodness of fit then drops when approaching the crossover between the power law scaling at short lengthscales and the flattening at large ones. Finally, when the fitting window covers only large lengthscales at which the configuration of the E–W line is completely flat, R^2 rises again. We note that since there are no fluctuations in our analytical data, at large lengthscales R^2 takes infinite values (there is no difference between the real data and the predicted data). We therefore forced the R^2 values to one instead of infinity to emphasise that in this interval there is a perfect fit by a power law with exponent 0.

Finally, an alternate procedure is to extract the roughness exponent ζ in reciprocal space, from the power law scaling of the structure factor $S(q)$

$$S(q,t) = \overline{\tilde{u}(q,t)\tilde{u}(-q,t)}, \tag{3}$$

where

$$\tilde{u}(q,t) = \frac{1}{L}\int dz\, u(z,t)\, e^{-iqz} \tag{4}$$

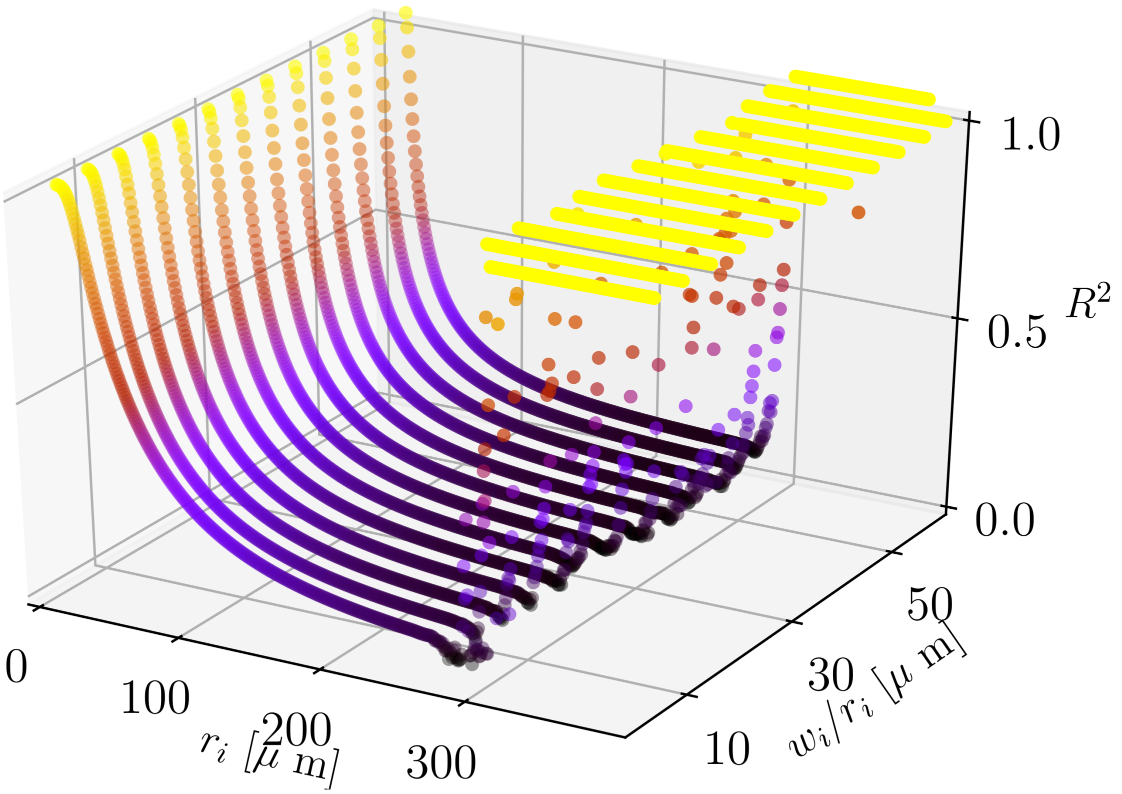

Figure 10. Our method to identify power law scaling regimes applied to an Edwards–Wilkinson elastic line. We show R^2 obtained for the fit of $\log B(r)$ given by Eq. 2. We detect only one power law scaling region with high R^2 at short lengthscales, and a second maximum of R^2 for at high lengthscales where the elastic line is flat (i.e. perfectly fitted by a power law with exponent 0).

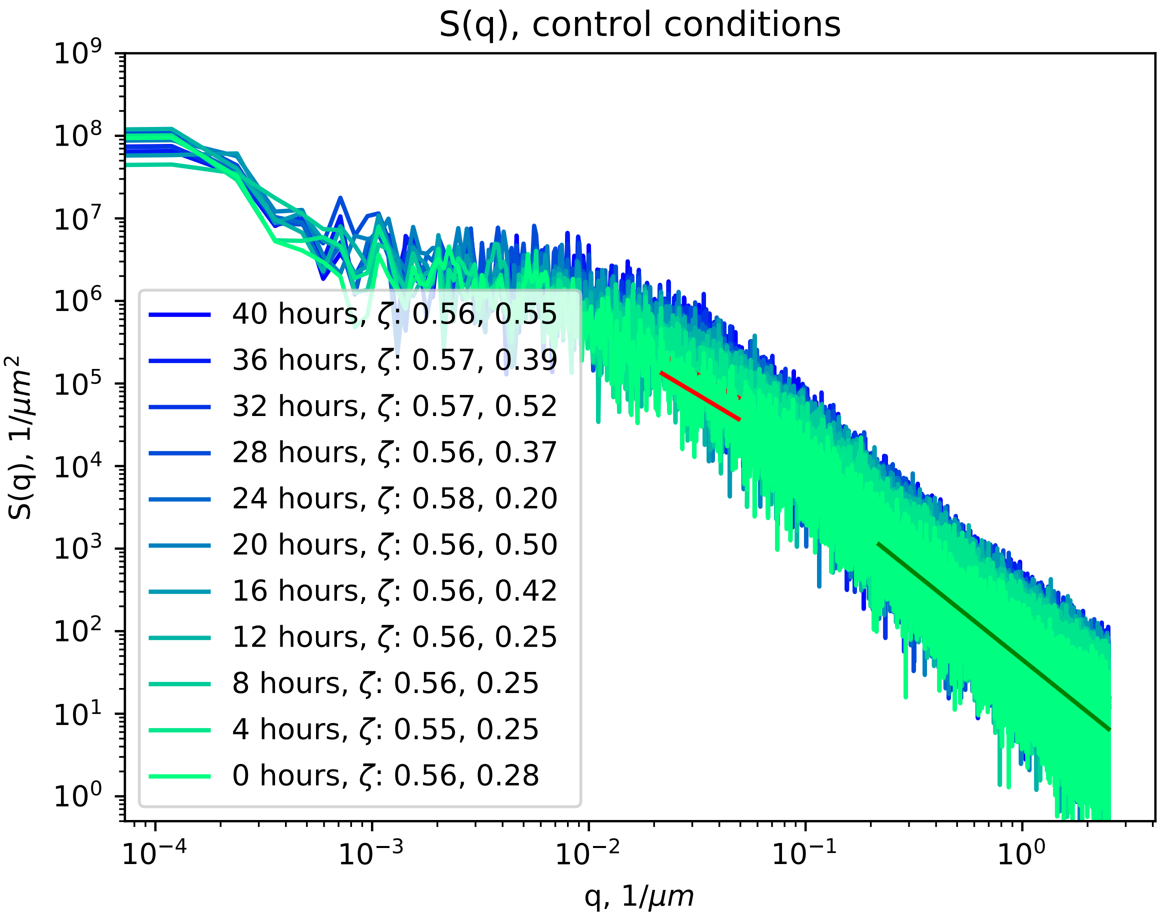

Figure 11. Evolution of the average structure factor $S(q)$ for the three Rat1 epithelial fronts under control conditions. Roughness exponent values are extracted from intervals corresponding to the real space regions I (subcell lengthscale, dark green) and II (few-cell lengthscales,red).

is the Fourier transform of the displacement field $u(z,t)$ defining the interface position. Formally, the structure factor $S(q)$ and the roughness correlation $B(r)$ contain the same geometrical information and are related through

$$B(r,t) = \int \frac{dq}{\pi} \left[1 - \cos(qr)\right] S(q,t). \quad (5)$$

While this approach is more prone to noise, where sufficient statistics are available, it provides generally more reliable estimates of ζ than the real space autocorrelation functions[7], and can fully address aspects of anomalous scaling not accessible via $B(r)$[2,8–12] In the structure factor, as shown in Fig. 11, we again observe two regions of power law scaling. Extracting the roughness exponent values from intervals corresponding to the real space regions I (subcell lengthscale, dark green) and II (few-cell lengthscales, red) we obtain $\zeta_I = 0.56 \pm 0.01$ and $\zeta_{II} = 0.29 \pm 0.08$, consistent with the values obtained from $B(r)$.

8 Probability distribution function of relative displacements

As a check of the nature of the interface and the symmetry of its relative displacements, we carried out a multiscaling analysis[13,14] to evaluate the probability distribution function (PDF) of relative displacements $\Delta u(r,z)$ and its characteristic scaling properties reflected in the behavior of its central moments[15]:

$$\sigma_n(r) = \overline{\langle |\Delta u(r)|^n \rangle} \sim r^{n\zeta_n}, \quad (6)$$

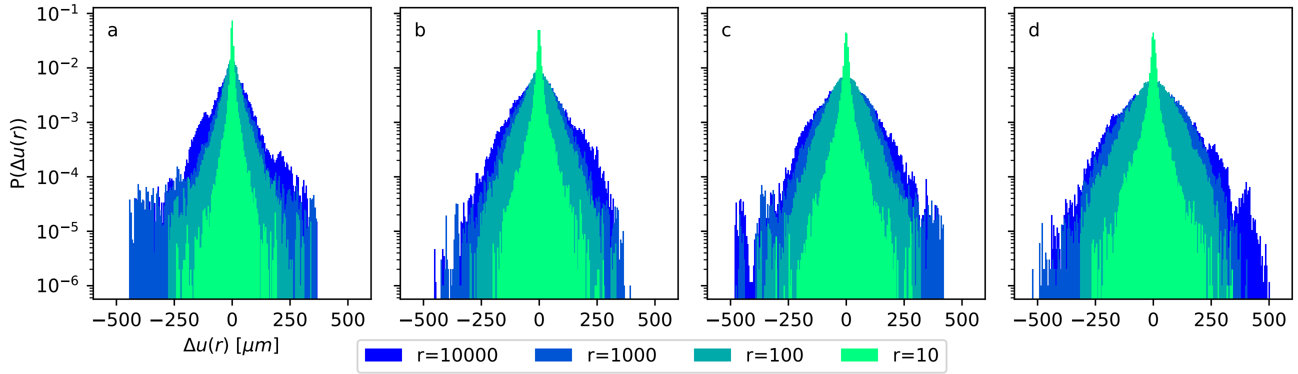

Figure 12. Probability distribution function of relative displacements $\Delta u(r,z)$ for the three Rat1 epithelial cell fronts under control conditions, shown for $r = 10$, 100, 1000, and 10000 µm at (a) 0, (b) 12, (c) 24m and (d) 44 hours after the removal of the silicone insert.

where ζ_n are the associated scaling exponents for the nth moment. For 1-dimensional monoaffine interfaces, the PDF is Gaussian[16–18], and the roughness function $B(r) \equiv \sigma_2(r) \sim r^{2\zeta}$ is sufficient to fully characterize the scaling, with a single-valued exponent $\zeta_n = \zeta \; \forall n$.

As shown in Fig. 12, the PDFs obtained for different r values and over the time of evolution of the front are highly symmetric for r above 100 µm, although the PDF at lowest $r = 10$µm values shows slight asymmetry. Consistent with the evolution of $B(r)$ discussed in the main paper, we see that for small lengthscales r, the PDF remains essentially unchanged with time. For larger lengthscales $r = 10000$, the initial PDF falls off from a Gaussian behaviour for small $\Delta u(r)$ to a steady background level, related to the effective flattening of the initial front configuration at high lengthscales. As the front evolves and roughens also at the higher lengthscales, the PDF for $r = 10000$ becomes more Gaussian.

The third (skewness) and fourth (kurtosis) moments show two regimes of power law scaling at subcell and few-cell lengthscales in regions I and II as defined in the main paper, from which the characteristic exponents ζ_3 and ζ_4 can be extracted. As shown in Figs. 13, 14, the value of the scaling exponents in region II appear to be universal, but diminish for region I.

9 Effective flattening by filling of cavities

During the measurements, we occasionally found large cavities at the edge of the colony, as shown in Fig. 15(a). The presence of these features significantly affected the dynamics of the colony, with cells rapidly moving towards the cavity and progressively filling it, as can be seen in Fig. 15(b,c), with the inflowing cells aligned radially towards the cavity center. Only when the cavity was filled did this section of the front continue to move outward again, as previously observed by Kim *et. al.*[19]. While we excluded affected segments of the fronts containing such features from the statistical analysis of roughness and dynamics, we believe that they are potentially related to the steady-state saturation of $B(r)$ at higher lengthscales.

10 Numerical simulations

The numerical simulations of proliferating cell fronts were performed by using the *epicell* package, developed at the University of Geneva[20], adapted to the modeling of mechanical properties of two-dimensional cell sheets[21,22].

The *epicell* model used is based on solving the following dimensionless Hamiltonian on a regular grid of two-dimensional hexagonal cells:

$$\overline{H} = \frac{1}{2}\sum_{cell\ \alpha}\left(\overline{A}_\alpha - 1\right)^2 + \frac{1}{2}\overline{\Gamma}\sum_{cell\ \alpha}\overline{L}_\alpha^2 - \overline{\Lambda}\sum_{edge\ e_{i,j}}\overline{L}_{i,j}$$

with $\overline{H} = \frac{H}{K\cdot(A^0)^2}$, $\overline{A}_\alpha = \frac{A_\alpha}{A^0}$, $\overline{\Gamma} = \frac{\Gamma}{K\cdot A^0}$, $\overline{L}_\alpha = \frac{L_\alpha}{A^0}$, $\overline{\Lambda} = \frac{\Lambda}{K\cdot(A^0)^{\frac{3}{2}}}$, $\overline{L}_{i,j} = \frac{L_{i,j}}{\sqrt{A^0}}$, H the Hamiltonian, A^0 the preferred cell area, K the cell area elasticity, Γ the cell perimeter contractility, Λ the inter-cell adhesion represented by the cell-cell edge tension, A_α the area of cell α, $L_{i,j}$ the length of the inter-cell edge $e_{i,j}$. The re-normalization of the Hamiltonian with the constraint that all cells are of the same fixed type, elasticity and preferred size, yields a two-variable system, with reduced parameters $\overline{\Gamma}$ and $\overline{\Lambda}$.

Additionally, the model was modified to include a non-trivial cell division descriptor, favoring divisions to occur just behind the cell front, in line with out empirical observations. This was implemented through setting a weight prefactor to the division probability following $W_{div} = 0$ at the cell front ($d = 0$) and $W_{div} = \exp -\frac{d-1}{3}$ for cells with distance d behind the front.

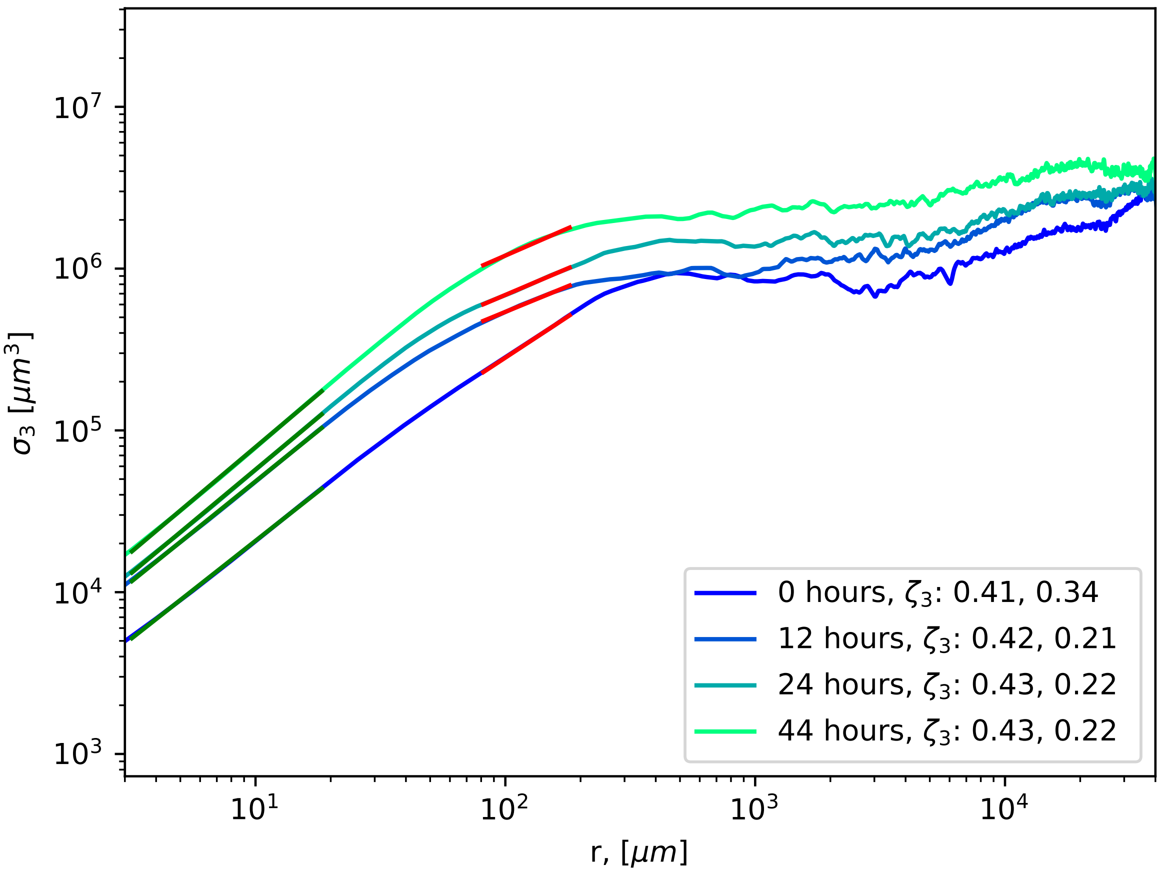

Figure 13. Power law scaling of the central moments of the PDF of relative displacements. Skewness with power law fits ($\sigma_3 r \sim r^{\zeta_3}$) of the subcell and few-cell lengthscale regions *I* and *II*.

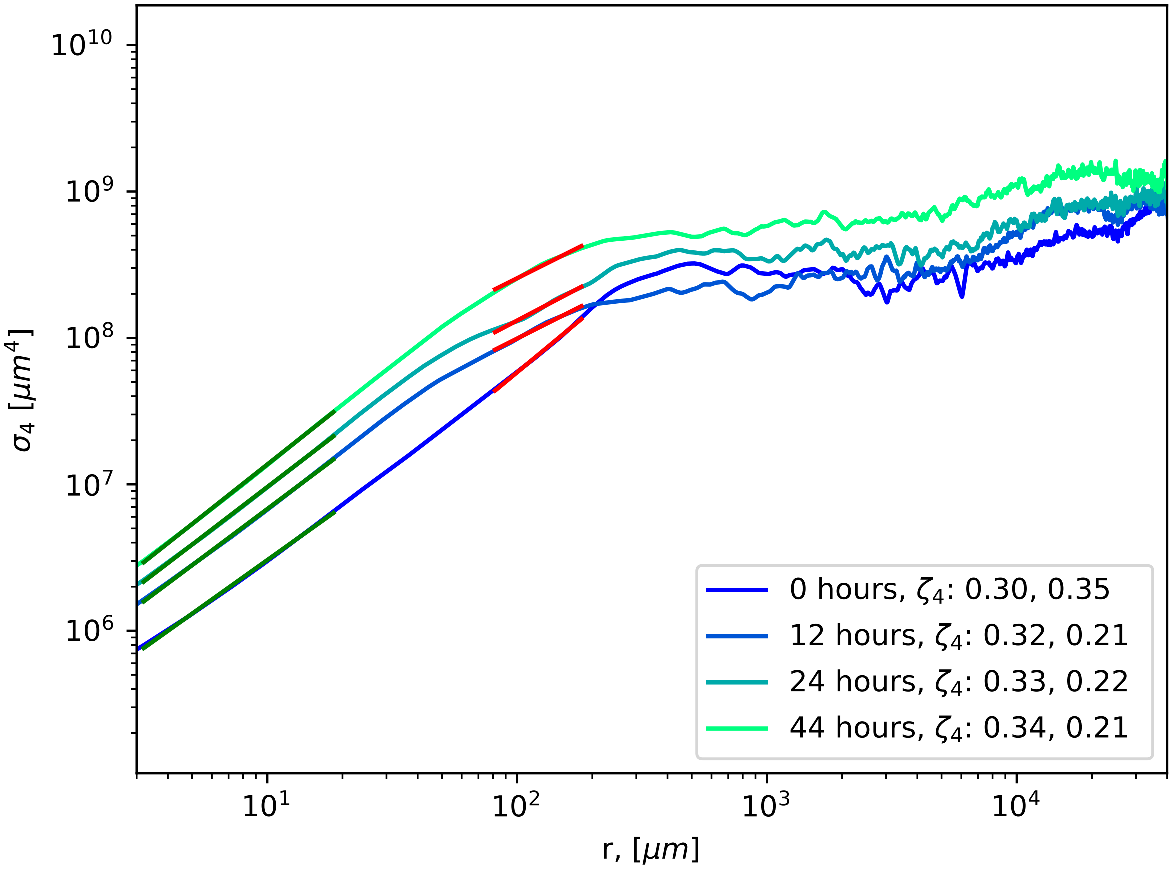

Figure 14. Power law scaling of the central moments of the PDF of relative displacements. Kurtosis with power law fits ($\sigma_4 r \sim r^{\zeta_4}$) of the subcell and few-cell lengthscale regions *I* and *II*.

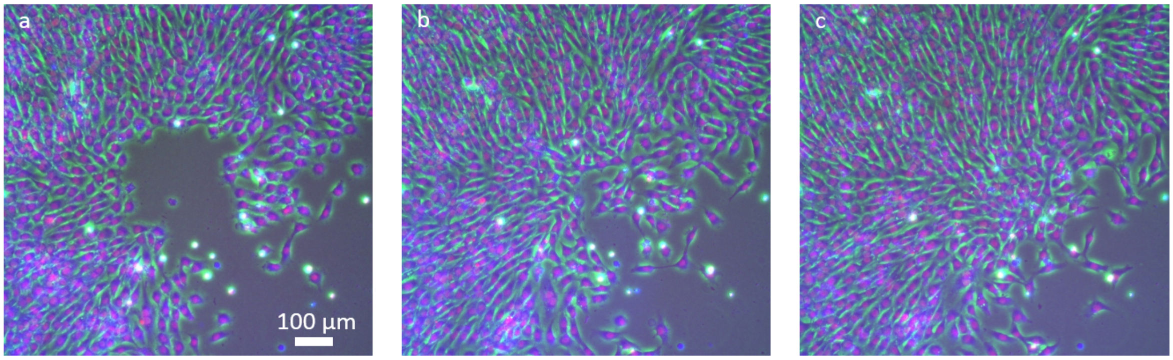

Figure 15. Cavity filling by collective/directed cell flow. Fluorescence images of a cell front, imaged every 4 hours, with a cavity initially present (a), progressively filled by the rapid inflow of cells from the bulk of the colony (b). Only upon complete filling of the cavity (c) does the front start to move outward again.

Parameters and execution

Seventeen parameters are necessary to run the *freeBoundary* application included in the *epicell* package. They are listed below with their description and values taken for the current study:

- **modelId**: The model dictates which cells are selected for division in priority. Here, set to 3, so that the largest and less compressed cells would divide first.
- **nRep**: Number of replicates of the cell front, here set to 1.
- **myseed**: Seed for the random number generator, here set to 1.
- **K**: The cell area elasticity parameter K. Set to $2.256 \cdot 10^9 N/m^3$ following previous studies on mechanical properties of cell sheets.
- **A0**: Preferred cell area A^0. Set to $3.14 \cdot 10^{-10} m^2$, following the experimental observations on the average size of the Rat1 cell line.
- **lambdaNorm**: Normalized inter-cell adhesion, $\overline{\Lambda}$. Parameter explored in phase space from -1.975 to 0.5 in steps of 0.025.
- **gammaNorm**: Normalized cell perimeter contractility, $\overline{\Gamma}$. Parameter explored in phase space from -0.3 to 0.675 in steps of 0.025.
- **ncols**: Number of columns of cells within the cell sheet. Set to 20 here to have a thin but long cell sheet.
- **nrows**: Number of rows of cells within the cell sheet. Set to 10^3 here to have a thin but long cell sheet.
- **xmin**: Left hard boundary position. Set to 0 here so that the system imposes boundaries automatically after the initial relaxation step.
- **xmax**: Right hard boundary position. Set to 0 here so that the system imposes boundaries automatically after the initial relaxation step.
- **ymin**: Bottom hard boundary position. Set to 0 here so that the system imposes boundaries automatically after the initial relaxation step.
- **nbDivMax**: The maximum number of cell divisions before the simulation terminates. Set here to 10^5 to allow a large number of divisions compared to the number of cells in the sheet.
- **elasticCoef**: Elastic coefficient of the hard boundaries, used in this work to mimic an infinite in-compressible cell sheet on three of the sides as to promote growth along one direction. Set to 10 here to provide a hard boundary.
- **sdLambda**: Standard deviation of the inter-cell adhesion Λ. Set to 0 in the current study.
- **sdGamma**: Standard deviation of the cell perimeter contractility Γ. Set to 0 in the current study.
- **boundaryLambda**: Normalized line tension of the cell sheet edges, used for simulating an interaction of the cells with the external media. Set to 0 in the current study.

The *freeBoundary* code was executed on the University of Geneva cluster, *Baobab*, with a time constraint before termination of 12 hours. The 4000 $\overline{\Lambda}, \overline{\Gamma}$ parameter pairs were computed in parallel and 881 simulations were successful. Unsuccessful simulations were either due to nonphysical parameters (1411 simulations), impossible convergence at the initial relaxation stage (1050 simulations), nonphysical mechanical behaviour of vertices (651 simulations), and finally unexpected simulation terminations due to specific pathological parameter sets (7 simulations). The outcome of all parameter pairs is shown in Fig. 16, with successful simulations labeled as *OK*.

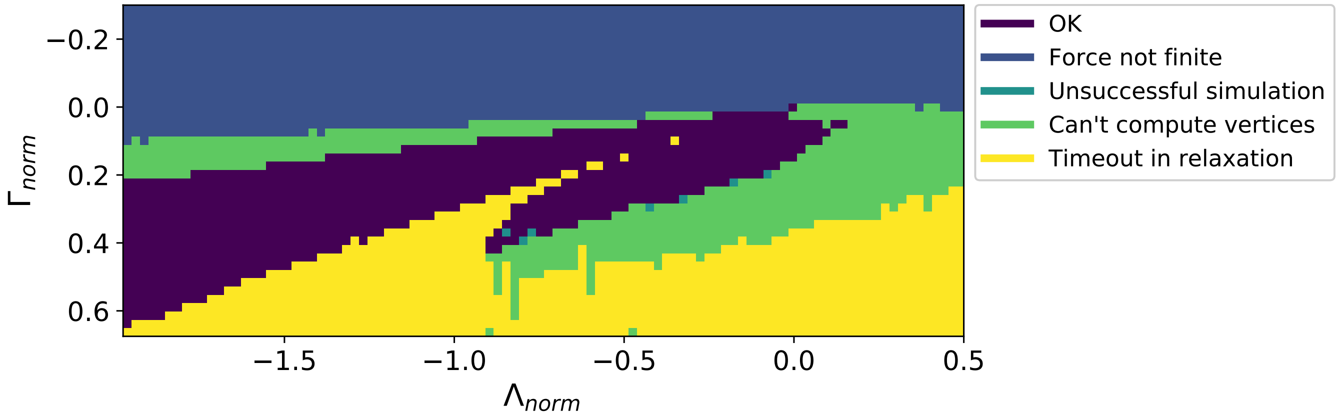

Figure 16. Complete simulation space with 4000 $\overline{\Lambda},\overline{\Gamma}$ parameter pairs. Out of this, 881 simulations were successful, labeled as *OK*. The unsuccessful simulations either occurred due to nonphysical parameters (1411 with infinite forces and 651 with nonphysical vertices) or due to non-convergence during the initial relaxation step (1050 simulations). A minority, 7 simulations, ended unexpectedly due to specific pathological parameter sets.

Phase diagram investigation

Our study focused on the successfully simulated region of the phase diagram, as shown in Fig. 17. The phase diagrams for the asymptotic roughness exponent ζ, quality of fit R^2 for the asymptotic exponent, total front displacement from its initial position, velocity of the front over the final 100000 iterations, and the number of iterations for each simulation are shown in Fig. 17(a,c,e,g,i) respectively.

As discussed in the main text, the analysis of simulation parameters enables the extraction of two branches of physical observables, with the lower branch exhibiting pathological behaviour including quasi-nonexistent front displacement/velocity, periodic features in the roughness, and large error during extraction of the roughness exponent ζ. Through the combination of the phase diagrams, a region of *stability* was defined visually as a red bounding polygon, with edges located at $(\overline{\Lambda},\overline{\Gamma}) = [(0,0.03),(0,0.06),(-1.975,0.49),(-1.975,0.27)]$. This filtering enables the removal of pathological scenarios, and allows for a better selection of the appropriate models for comparison with *in-vitro* experiments. For instance, the overall average roughness exponent $\overline{\zeta} = 0.69$ as shown in the distribution Fig. 17(b) is highly asymmetric, whereas sampling ζ values in the region of stability yields an average roughness exponent $\overline{\zeta} = 0.74$ with a smaller error and a more symmetric distribution.

It is also noteworthy that whilst ζ and the number of iterations following the initial relaxation step (Fig. 17(i,j)) seem to be concentrated around specific values, the total displacement and final velocity in Fig. 17(e,f,g,h) are more evenly distributed, and in fact tend to increase with decreasing inter-cell adhesion and cell contractility.

References

1. Guyonnet, J. *Growing up at the nanoscale: studies of ferroelectric domain wall functionalities, roughening, and dynamic properties by atomic force microscopy*. Ph.D. thesis, DQMP, University of Geneva (2013). DOI: 10.13097/ARCHIVE-OUVERTE/UNIGE:31526.
2. Guyonnet, J., Agoritsas, E., Paruch, P. & Bustingorry, S. Statistics of domain wall roughness: A survey of different scaling analyses. *Arxiv.org* (2019).
3. Bathany, C., Beahm, D., Felske, J. D., Sachs, F. & Hua, S. Z. High throughput assay of diffusion through Cx43 gap junction channels with a microfluidic chip. *Anal. Chem.* **83**, 933–939, DOI: 10.1021/ac102658h (2011).
4. Trepat, X. *et al.* Physical forces during collective cell migration. *Nat. Phys.* **5**, 426–430, DOI: 10.1038/nphys1269 (2009).
5. Kolton, A. B., Rosso, A. & Giamarchi, T. Relaxation of a flat interface. *Phys. Rev. Lett.* **95**, 180604 (2005).
6. Caballero, N., Agoritsas, E., Lecomte, V. & Giamarchi, T. From bulk descriptions to emergent interfaces: Connecting the Ginzburg-Landau and elastic-line models. *Phys. Rev. B* **102**, 104204 (2020).
7. Schmittbuhl, J., Vilotte, J.-P. & Roux, S. Reliability of self-affine measurements. *Phys. Rev. E* **51**, 131 (1995).
8. Ramasco, J. J., López, J. M. & Rodriguez, M. A. Generic dynamic scaling in kinetic roughening. *Phys. Rev. Lett.* **84**, 2199 (2000).

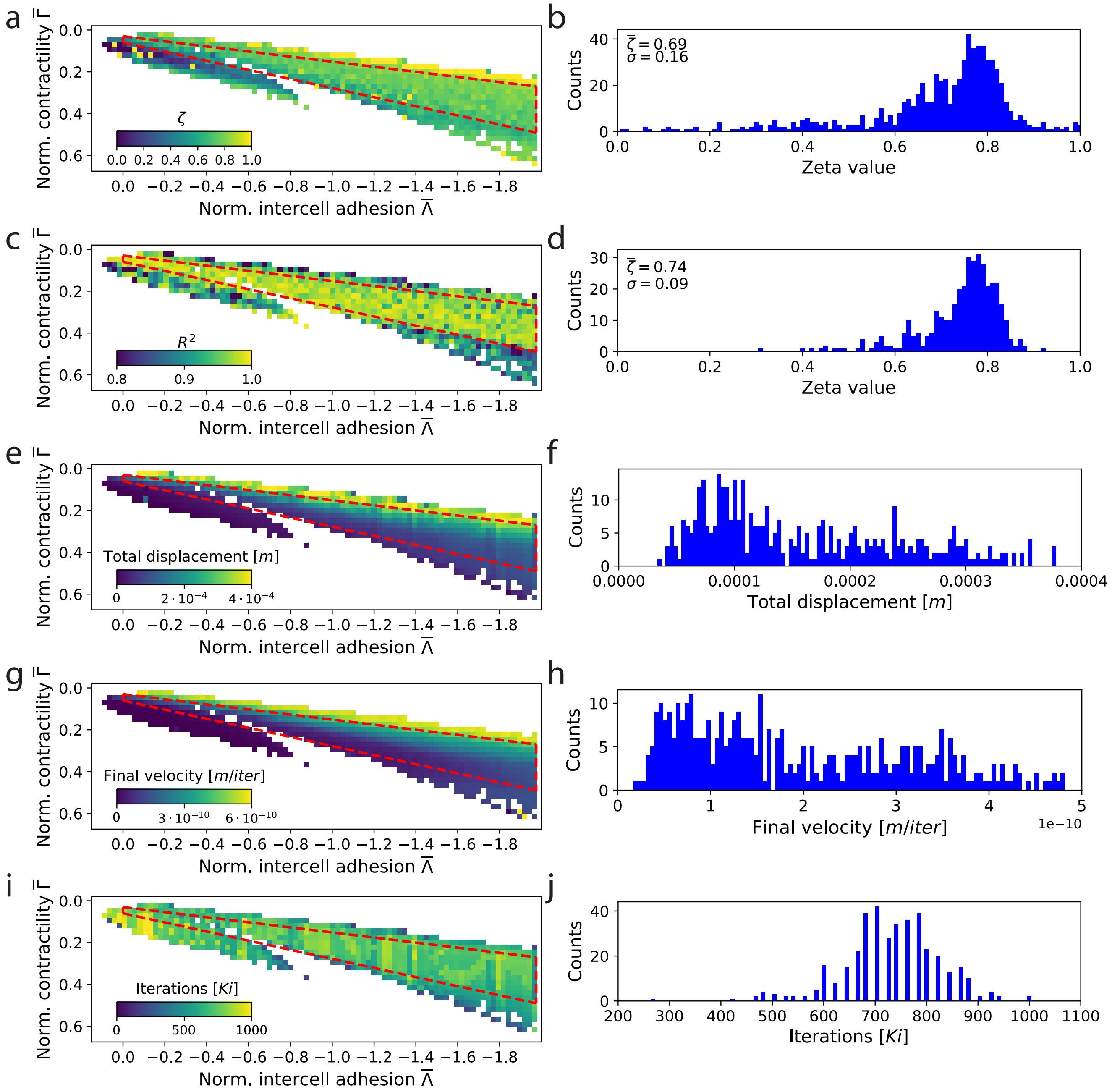

Figure 17. Simulation observable phase diagrams and their corresponding distributions. The red polygons delimit the region of stability defined from visual observation of the extracted physical parameters. (a) Phase diagram of the asymptotic roughness exponent ζ, computed by fitting the time evolution of the roughness exponents of fronts. (b) Distribution of the ζ values, showing a large spread, asymmetry and a mean at $\overline{\zeta} = 0.69$. (c) Goodness-of-fit estimate R^2 for the ζ fits, demonstrating a large region of high quality fits within the region of stability. (d) Distribution of ζ values within the region of stability, with a mean of $\overline{\zeta} = 0.74$. (e) Phase diagram and (f) region of stability distribution of the total front displacement. (g) Phase diagram and (h) region of stability distribution of the final front velocity. (i) Phase diagram and (j) region of stability distribution of the number of iterations for each simulation parameter pair following initial relaxation.

9. Ferrero, E. E., Bustingorry, S. & Kolton, A. B. Nonsteady relaxation and critical exponents at the depinning transition. *Phys. Rev. E* **87**, 032122, DOI: 10.1103/PhysRevE.87.032122 (2013).

10. Bustingorry, S., Kolton, A. & Giamarchi, T. Thermal rounding of the depinning transition in ultrathin Pt/Co/Pt films. *Phys. Rev. B* **85**, 214416 (2012).

11. Agoritsas, E., Lecomte, V. & Giamarchi, T. Temperature-induced crossovers in the static roughness of a one-dimensional interface. *Phys. Rev. B* **82**, 184207, DOI: 10.1103/PhysRevB.82.184207 (2010).

12. Agoritsas, E., Lecomte, V. & Giamarchi, T. Static fluctuations of a thick one-dimensional interface in the 1+1 directed polymer formulation: Numerical study. *Phys. Rev. E* **87**, 062405, DOI: 10.1103/PhysRevE.87.062405 (2013).

13. Santucci, S. *et al.* Statistics of fracture surfaces. *Phys. Rev. E* **75**, DOI: 10.1103/physreve.75.016104 (2007).

14. Guyonnet, J., Agoritsas, E., Bustingorry, S., Giamarchi, T. & Paruch, P. Multiscaling analysis of ferroelectric domain wall roughness. *Phys. Rev. Lett.* **109**, DOI: 10.1103/physrevlett.109.147601 (2012).

15. Agoritsas, E., Lecomte, V. & Giamarchi, T. Disordered elastic systems and one-dimensional interfaces. *Phys. B: Condens. Matter* **407**, 1725–1733, DOI: 10.1016/j.physb.2012.01.017 (2012).

16. Halpin-Healy, T. Directed polymers in random media probability distributions. *Phys. Rev. A* **44**, R3415 (1991).

17. Mézard, M. & Parisi, G. Replica field theory for random manifolds. *J. de Physique I* **1**, 809 (1991).

18. Rosso, A., Santachiara, R. & Krauth, W. Geometry of gaussian signals. *J. Stat. Mech.* L08001 (2005).

19. Kim, J. H. *et al.* Propulsion and navigation within the advancing monolayer sheet. *Nat. materials* **12**, 856–863 (2013).

20. Merzouki, A., Malaspinas, O. & Chopard, B. *EpiCells Project* (2017). http://epicells.unige.ch/.

21. Merzouki, F. A. *Numerical Modelling of Confluent Cell Monolayers : Study of Tissue Mechanics and Morphogenesis*. Ph.D. thesis, Université de Genève (2018). DOI: 10.13097/ARCHIVE-OUVERTE/UNIGE:106434.

22. Merzouki, A., Malaspinas, O. & Chopard, B. The mechanical properties of a cell-based numerical model of epithelium. *Soft Matter* **12**, 4745–4754, DOI: 10.1039/c6sm00106h (2016).
